# Real-World Evidence Shows Gaps in Awareness, Medical Help-Seeking, and Diagnosis for Primary Dysmenorrhea but Not Premenstrual Syndrome: Cross-Sectional Observational Study

**DOI:** 10.2196/68148

**Published:** 2025-09-11

**Authors:** Matthias Roos, Verena Wimmelbacher, Lisa Klein, Marija Kesić, Ann-Katrin Rueß, Christina Necker, Nicole Mähler, Petra Stute, Christoph Abels, Tobias Kruse

**Affiliations:** 1 SubjectWell, Denninger Str. 30Munich, 81679, Germany, +49 89 215 37 4990; 2 Bionorica SE, Neumarkt in der Oberpfalz, Germany; 3 Department of Obstetrics and Gynecology, University Clinic Inselspital, Bern, Switzerland

**Keywords:** real-world evidence, RWE, real-world data, RWD, HEOR, patient recruitment, digital patient recruitment, awareness, clinical trial, dysmenorrhea, primary dysmenorrhea, premenstrual syndrome, PMS, menstrual pain, menstrual cramps, Health Economics and Outcomes Research

## Abstract

**Background:**

Menstrual complaints are widespread but often stigmatized. The most common is dysmenorrhea, or menstrual cramps, which manifests as mild to severe pain during menstruation and affects >40% of women throughout their reproductive lifespan. Dysmenorrhea is often endured silently or managed through self-medication. Consequently, a vast majority of patients with dysmenorrhea may not be found in medical practices, highlighting the need for direct-to-patient communication to reach a broad and diverse patient population.

**Objective:**

Primarily, this study aims to reveal the diagnosis status, pain levels, comorbidities, eligibility, and willingness to participate in clinical trials of women affected by dysmenorrhea and menstrual discomfort, based on a broad patient population not necessarily reached in medical practices. Second, this study attempts to test the effectiveness of direct-to-patient communication via online campaigns in engaging patients affected by dysmenorrhea or conditions that may benefit from direct-to-patient communication.

**Methods:**

Women experiencing menstrual pain were reached through a targeted online campaign using Google Ads (Google LLC) and Facebook (Meta Platforms, Inc) in Germany, Austria, and Poland and were surveyed from April to June 2023. This study is observational.

**Results:**

We surveyed 3546 women, 94.6% (3230/3413) of whom reported symptoms consistent with dysmenorrhea, highlighting the high specificity of the Google and Facebook campaigns. Of the affected women in Germany and Austria, 88.5% (874/988) reported pain levels of 6 or higher on a scale of 0 to 10, with even higher pain levels observed in Poland. Elevated pain levels were correlated with dysmenorrhea symptoms but not with premenstrual syndrome (PMS) symptoms. Notably, of the 3230 women reporting symptoms consistent with dysmenorrhea, only 4.6% (n=149) reported being diagnosed with the condition, regardless of elevated pain levels. This can be attributed to two factors: (1) 90.3% (3065/3395) of surveyed women did not seek medical advice, were uncertain about their diagnosis, or their menstrual-related symptoms were not recognized as pathological and (2) among the 9.7% (330/3395) diagnosed, only half of DYS-affected women (149/318, 46.9%) were diagnosed with dysmenorrhea. The other 53.1% (169/318) were diagnosed with PMS but not dysmenorrhea despite regularly experiencing dysmenorrhea symptoms. The situation was better for PMS. Among the 330 diagnosed women, 77.3% (n=255) were diagnosed with PMS, in line with the 80.1% (2729/3409) PMS prevalence in the survey population. Overall, about 8.6% (235/2729) of women with PMS symptoms reported having been diagnosed with PMS, nearly double the diagnosis rate reported for dysmenorrhea.

**Conclusions:**

The data reveal a significant diagnostic gap for dysmenorrhea, but not necessarily for PMS, even in high-income countries, as observed in Germany, Austria, and Poland. In these 3 countries, most dysmenorrhea-affected women do not seek medical advice, and up to half of dysmenorrhea diagnoses might be missed. Thus, most affected women might not be found in medical settings (doctors’ offices and clinics) despite experiencing significant pain. Online campaigns are shown to effectively reach individuals with menstrual complaints, including those who are undiagnosed or not seeking medical care.

## Introduction

### Dysmenorrhea Prevalence and Key Characteristics

Menstrual discomfort is widespread, varies in severity, and affects the quality of life (QoL) of those impacted. Around 17%-81%, and potentially even up to 97%, of women experience menstrual pain, according to a review by the World Health Organization (WHO) [1]. In a 2017 internet-based survey in the Netherlands, 14% of 14- to 45-year-old women reported absenteeism from work or school during their menstrual periods, with more than 3% reporting this for every or almost every menstrual cycle [2]. Pain levels can be particularly high in cases of menstrual cramps, referred to as primary dysmenorrhea if there is no underlying pathology (eg, endometriosis and myoma). Underlying conditions lead to the diagnosis of secondary dysmenorrhea. Dysmenorrhea is one of the most common conditions affecting women of reproductive age, with current literature reporting prevalences from 45% to 95% among menstruating individuals [3,4]. A survey of more than 42,800+ Dutch women by Schoep et al [5] revealed a dysmenorrhea prevalence of 85%, making dysmenorrhea the most common menstrual symptom in their survey. Armour et al [6] found a dysmenorrhea prevalence of 71%, based on a literature review that included 21,500+ young women. In 2022, analyzing patients with menstrual cyclic disorders who were prescribed Vitex agnus-castus dry extract BNO 1095 (Agnucaston), Höller et al [7] found that 44% of them were affected by dysmenorrhea. Dysmenorrhea is also characterized by a specific timing pattern: symptoms typically start a few hours before or with the onset of menstruation and subside as bleeding ends, with pain peaking during the first 24‐36 hours [8]. Primary dysmenorrhea additionally requires onset with adolescence, normal physical examination, and absence of pelvic abnormalities. Contributing factors are complex; the pathomechanism is not fully understood [9-11]. Dysmenorrhea can cause long-term QoL impairments, impacting mental and physical well-being, relationships, and career outcomes, while reinforcing hyperalgesic priming and increasing the risk of chronic pelvic pain [12]. Treatment commonly involves patient education and reassurance [13] and prescribing nonsteroidal anti-inflammatory [4,14,15] medication or combination therapies [14] to relieve experienced pain. However, 18% of women experiencing dysmenorrhea remain unresponsive to these measures [10].

### Background and Objectives of This Study

Viewing menstrual discomfort and associated pain as normal, interpreting it as a personal mood, and stigma surrounding menstruation, including also social embarrassment, can lead affected women to not seek professional advice from general practitioners or gynecologists [16-18]. Primary dysmenorrhea is considered one of the most underdiagnosed conditions [13]. In addition, affected individuals may mistake dysmenorrhea for premenstrual syndrome (PMS, which occurs 5‐10 days before menstruation, not during it). Self-care, both pharmaceutical and nonpharmaceutical, is common [19], but the most effective options for pain management are not necessarily chosen [20]. Fewer than half of young women achieve satisfactory pain relief from self-care analgesics, most commonly paracetamol [20]. For these reasons, a vast majority of patients with dysmenorrhea endure dysmenorrhea silently, and those finally diagnosed with dysmenorrhea might not inherently represent the diverse spectrum of women affected by the condition. Thus, direct-to-patient communication can be highly beneficial to effectively reach the respective patient population, especially for clinical trial recruitment. Accordingly, Bionorica SE partnered with Trials24 GmbH, now part of SubjectWell, Inc, to reach patients with (primary) dysmenorrhea online, leveraging awareness campaigns via common social media.

This study aims to assess the diagnosis status, pain severity, comorbidities, and clinical trial eligibility of women with dysmenorrhea and menstrual discomfort, focusing on a broad patient population not typically reached in medical settings. In addition, it evaluates the effectiveness of direct-to-patient outreach via online campaigns (Google Ads and Facebook) in engaging individuals with dysmenorrhea or related conditions who may benefit from such an approach.

The insights below were collected while assessing the feasibility of online channels for patient recruitment for a randomized clinical trial (European Union Clinical Trial number 2023-503688-41) evaluating the efficacy of a *Vitex agnus castus*–containing herbal medicinal product in primary dysmenorrhea, and to generally validate if online channels are well-suited for effectively reaching women affected by (primary) dysmenorrhea. In the following, we present our findings from the associated patient awareness campaign and online survey.

## Methods

### Campaign Implementation

To create real-world data, patient surveys were conducted in Germany and Austria from April 24 to May 7, 2023, and in Poland from May 22 to June 4, 2023, each covering 14 days. Germany, Austria, and Poland were selected due to a planned clinical trial on menstrual pain in these countries, making them practical examples of direct-to-patient communication in clinical research. Survey awareness was generated through campaigns on Facebook and Google, reaching patients directly rather than through doctors’ offices and hospitals. Advertisements were designed to be compliant, simple to understand, and to reach a broad audience. Visuals and texts used in the awareness campaign are displayed in the supplementary material, Multimedia Appendix 1. The results presented herein are observational.

### Patient Questionnaire

Participants were not required to have previous knowledge of dysmenorrhea or PMS. Instead, the questionnaire addressed symptoms such as menstrual pain and discomfort, considering their timing relative to the menstrual cycle. All individuals who responded to the campaign were included in the analysis, though subpopulations were formed to compare proportions. There were no inclusion or exclusion criteria for completing the questionnaire, other than answering the currently displayed questions to proceed. All participants were asked all questions, regardless of their previous answers (ie, no population filtering in the questionnaire), except for 2 case-dependent questions on contraception and contact data sharing. Questions on contact data were displayed only after participants expressed interest in receiving further information about new clinical studies in the field of menstrual pain and only if they provided explicit opt-in following an additional privacy statement. Sharing contact data remained voluntary and in compliance with the General Data Protection Regulation. To maximize completion rates, brevity of the questionnaire was prioritized over collecting additional information. To view the questionnaire, please refer to Multimedia Appendix 2. Some questions (eg, contraception) were used to estimate the proportion of individuals potentially eligible for clinical trial participation in the field of menstrual pain and can be found again in Multimedia Appendix 2 for qualifying responses. Pain levels were assessed as the maximum intensity of menstrual pain on a 0‐10 scale, where 0 equals no pain or cramps, and 10 equals very severe pain or very severe cramps. As noted above, none of the above aspects led to exclusion from continuing the survey. Subpopulations were created solely during the analysis of survey results (see below), based on participants’ responses.

### Data Analysis

Data analysis focused on comparing proportions and the intercorrelation between variables, using Cramer V (for nominal variables, based on chi-square statistics) and Spearman rank correlation coefficient (for ordinal variables, revealing rank correlations). Subgroups were generated using both contingency tables and by filtering and counting individuals who met the conditions specified in the associated text or figure. Automated processes were validated through various manual spot checks. Missing data from unanswered questions were excluded during the data evaluation of the respective subpopulation, resulting in intrinsic patient response uncertainty of 0.1%‐0.7% per health-situation question, as the trend for these individuals has remained unclear (for instance, an unknown trend toward “yes” in a binary question among those who did not answer the question). See Multimedia Appendix 2 for per-question data completeness and dropouts. Costs per individual reached and per eligible contact were calculated by dividing the number of these individuals by the marketing budget, excluding and including donations. Cramer V (intercorrelation strength or “effect size”), denoted as V, was used to assess the following intercorrelations: occurrence of PMS symptoms versus dysmenorrhea symptoms; PMS/dysmenorrhea symptoms versus PMS/dysmenorrhea diagnosis received; occurrence of dysmenorrhea/PMS symptoms versus pain ≥6; pain ≥6 versus diagnosis received; dysmenorrhea/PMS symptoms versus medication usage; pain ≥6 versus medication usage; pain ≥6 versus willingness to participate in a clinical trial; willingness to participate in a clinical trial versus interest in further information. Cramer V correlation strength was interpreted as follows: 0.00≤V≤0.10: no or very weak; 0.10<V≤0.15: weak; 0.15<V≤0.30: moderate; 0.30<V≤0.5: strong; V>0.5: very strong. While the results of Cramer V analyses are presented in the Results section, associated data are summarized in Multimedia Appendix 3. Spearman rank correlation coefficients were used to assess the relationship between experienced pain (ranked by pain intensity, descending order) and questionnaire dropouts (ranked according to the question at which an individual dropped out, descending order). Calculated rank correlation coefficients, denoted as ρ, were interpreted as a negligible correlation if ρ≤0.1. Raw data can be accessed in Multimedia Appendix 4.

### Interpretation of Symptoms

To differentiate between dysmenorrhea-related and PMS-related symptoms, we relied on the timing of the symptoms relative to the menstrual cycle. We refer to dysmenorrhea symptoms as regular pain occurring from 1 day before to up to 3 days during menstrual bleeding, and primary dysmenorrhea if the pain was additionally reported to have begun during adolescence. The former is assessed through the question, “Do you regularly experience pain shortly before or during your period? (1 day before and up to 3 days during menstruation)”. Similarly, a positive response to “Do you regularly experience mood swings, pain, or general discomfort 5 to 10 days before your period?” was considered suggestive of PMS (“PMS symptoms”). PMS diagnoses typically require premenstrual symptoms in each of the 3 previous menstrual cycles [21], whereas the questionnaire captures symptoms experienced on a regular basis. This approach prioritized overall symptom burden over short- or mid-term snapshots at the expense of diagnosis precision: a survey of 2863 French women revealed significant fluctuation in their PMS status, with only 36% of those diagnosed with PMS continuing to meet the criteria a year later [22]. We acknowledge the residual uncertainty of the above procedure and therefore refer to symptoms indicative of dysmenorrhea or PMS, rather than definitively stating these diseases. Consequently, all statements—including those on potential underdiagnosis—pertain to the phenomenological symptoms or the discrepancy between reported symptom prevalence and diagnosis rates. Phrases such as “dysmenorrhea/PMS symptoms” are used as a concise way to refer to “symptoms related to dysmenorrhea/PMS” or “symptoms suggestive of dysmenorrhea/PMS” to improve readability. To differentiate between symptoms suggestive of primary versus secondary dysmenorrhea, we also asked about comorbidities including endometriosis, ulcerative colitis, Crohn disease, chronic bladder inflammation, past surgeries (that may cause current pain, adhesions, or abdominal scarring), and also queried if the pain onset was during adolescence. We do not distinguish between PMS and premenstrual dysphoric disorder (a severe version of PMS [21,23,24]), nor did the questionnaire include specific questions to differentiate between them. Instead, the questionnaire addressed pain intensities, diagnosis status, accompanying conditions, and key aspects relevant to clinical trial eligibility.

### Gender Diversity

We acknowledge that not all people who menstruate identify as women. In this study, we use the term “women” to refer to biological sex for simplicity. Both the Google and Facebook advertising algorithms were optimized for women aged 18‐49 years, with one of the multiple Facebook headlines specifically addressing “women with menstrual pain.” This approach reduced inclusivity in the awareness campaign in favor of algorithm optimization and cost efficiency, although the description in each ad used gender-neutral language.

### Ethical Considerations

Ethics committee approval was not required for this study, as it did not involve direct advertising of a clinical trial. No medical products were promoted. General ethical guidelines were followed, with the awareness campaign focusing on research interest, without claiming any patient benefits. Advertisements were designed with a neutral tone, focusing on menstrual discomfort and a willingness to help, while avoiding overly emotional language. Examples of Google Ads or Facebook headlines include “Together against [period pain | PMS | dysmenorrhea],” “Suffering from [period pain | PMS | dysmenorrhea]?,” “Frequent [period pain | PMS | dysmenorrhea]?,” and “Women with [menstrual cramps | PMS | dysmenorrhea] wanted. Help now.” These were displayed in German and Polish for Germany/Austria and Poland, respectively. The advertisements also stated that €3 (Germany/Austria; approximately US $3.27) or zł13 (Poland; approximately €3 or US $3.27) would be donated to a women’s health organization for each completed questionnaire, with the organization selected by respondents at the end of the survey. A total of 687 cases in Germany/Austria and 1468 cases in Poland qualified for donations, totaling €2061 (approximately US $2246) and zł19,084 (approximately €4461 or US $4862), respectively, with the donations covered by SubjectWell. See Multimedia Appendix 1 for additional information and visuals. Survey participants were additionally provided with background information (including the survey’s scope, motivation, responsible party, and contact details) as well as data protection and cookie information (“privacy policy”). Study participants needed to consent to our privacy policy before they could share personally identifiable information (PII). If the study participants decided against leaving their PII, the survey remained technically anonymous for them. All insights in this study rely on anonymous, deidentified data.

## Results

### Campaign Efficiency and Specificity

**Figure 1.**
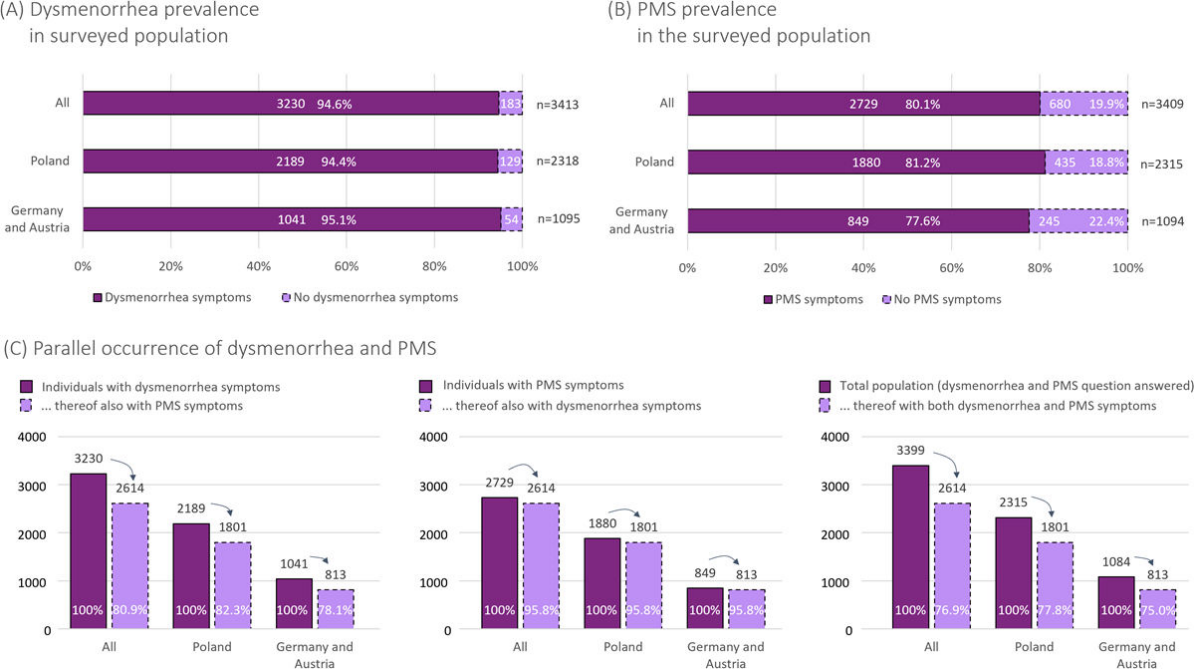
Specificity of the menstrual pain campaign. (A) Dysmenorrhea symptom frequency among the population reached by the online campaign. (B) PMS frequency in the reached population. (C) Overlap between dysmenorrhea and PMS. PMS: premenstrual syndrome.

The surveys reached a total of 3546 participants, with 1154 individuals from Germany/Austria and 2392 from Poland. Among them, 94.2% (3342/3546) completed all questions, and 92.5% (3188/3448) were women aged 18-49 years. Patients experiencing dysmenorrhea symptoms were reached with a 94.6% success rate: out of the 3413 women answering the question on their menstrual pain situation, 3230 regularly experienced pain 1 day before and up to 3 days during menstruation, suggesting dysmenorrhea (Figure 1A). The dysmenorrhea symptom rates among surveyed women were essentially the same for Germany/Austria (1041/1095, 95.1%) and Poland (2189/2318, 94.4%), with campaign texts provided in the native language. Of the surveyed women with dysmenorrhea symptoms, 78.8% (2545/3230) have experienced menstrual pain since their youth (within 2 years after the onset of menstruation), suggesting primary dysmenorrhea (Table 1). This percentage also includes women who were uncertain about when their symptoms began. Moreover, 74.1% (2394/3230) of surveyed women with dysmenorrhea symptoms had both: symptoms that began during adolescence and a normal menstrual cycle of 24‐38 days [25,26]. Alongside dysmenorrhea symptoms, 80.1% (2729/3409) of the surveyed women (Germany/Austria: 849/1094, 77.6%; Poland: 1880/2315, 81.2%; Figure 1B) experienced symptoms consistent with PMS. Notably, 95.8% (2614/2729) of the women who reported PMS symptoms also experienced dysmenorrhea symptoms (Figure 1C), so that 76.9% (2614/3399) of all surveyed women reported being affected by both dysmenorrhea and PMS symptoms. Irrespective of the high prevalence of both dysmenorrhea and PMS in the surveyed population, the association between dysmenorrhea symptoms and PMS symptoms was weak (Cramer V=0.100).

**Table 1.** Number of individuals with dysmenorrhea symptoms, including onset in adolescence and menstrual cycles of 24‐38 days.

Characteristic	Germany/Austria, n (%)		Poland, n (%)		All, n (%)	
Dysmenorrhea symptoms	1041 (100)		2189 (100)		3230 (100)	
Starting in adolescence	783 (75.2)		1762 (80.5)		2545 (78.8)	
With a menstrual cycle of 24 to 38 days	740 (71.1)		1654 (75.6)		2394 (74.1)	

### Patient Engagement and Data Completeness

Of the 3546 individuals who opened the questionnaire, 3342 (94.2%) completed all health-related questions, while only 204 dropped out. Among the 204 dropouts, nearly half (98 individuals) left before answering the first question. The completion rate among those who started filling out the questionnaire (ie, answered the first question) was as high as 96.9% (3342/3448). After the first 3 questions, dropouts ranged from 1 to 8 people per question, equaling less than 0.1%‐0.2% of survey participants per question, except for questions on contraception (0.4% dropout rate). See Multimedia Appendix 2 for further details and a flow chart visualizing both dropouts and the questionnaire completion rate on a question-by-question basis. Pain intensities were addressed in question 5. For those who had already completed questions #1–#5, there was no association between pain intensity and completing the remaining questionnaire (versus dropping out), as indicated by a Spearman rank correlation coefficient of just 0.035.

### Pain Levels

**Figure 2.**
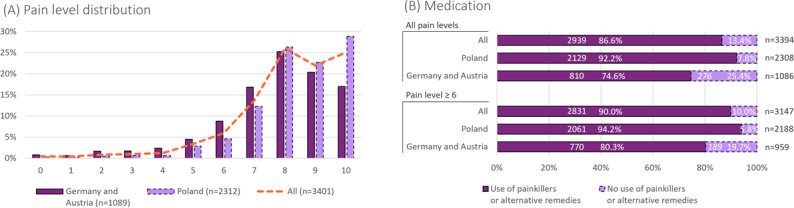
Pain levels and medication use for pain relief in the survey population. (A) Menstrual pain intensity measured on an 11-point scale (0=no pain; 10=maximum pain). Occurrence rates increase with pain levels and plateau at pain levels of 8 or higher (dashed line). (B) Medication usage for pain relief.

Affected women were asked to rate the maximum intensity of their menstrual pain on a numeric rating scale from 0 to 10, with 10 corresponding to very severe pain or very severe cramps (Figure 2A). In Germany/Austria, about 88.5% (874/988) of affected women have endured pain levels of 6 or higher, and over 62.0% (613/988) reported levels of 8 or higher. The average maximum pain intensity was 7.7. In Poland, reported pain levels were even higher: 94.8% (2192/2312) reported pain intensities of 6 or higher, with about 77.9% (1802/2312) experiencing pain as strong as 8 or higher, and an average of 8.4. Cramer V revealed a moderate-to-strong association between dysmenorrhea symptoms and pain levels of 6 or higher (V=0.299), whereas PMS symptoms showed no or very weak association (V=0.075) with elevated pain. This trend did not change for pain levels of 8 or higher (see Multimedia Appendix 3). To deal with their menstrual pain, 74.6% (810/1086) of survey participants in Germany/Austria and 92.2% (2129/2308) in Poland made use of pain medication or alternative remedies (Figure 2B). The surveyed population predominantly included individuals with high pain levels; therefore, restricting the statistics to those with pain levels≥6 does not significantly alter the results (Figure 2B). As might be expected, there is a strong correlation between medication usage and elevated pain (V=0.352 for pain ≥6). Elevated pain was found to be strongly linked to dysmenorrhea symptoms, but only marginally to PMS symptoms (see above). Consistently, we found a moderate association between medication usage and dysmenorrhea symptoms (V=0.213), but only a weak association with PMS symptoms (V=0.057).

### General Diagnosis Status (Entire Survey Population)

**Figure 3.**
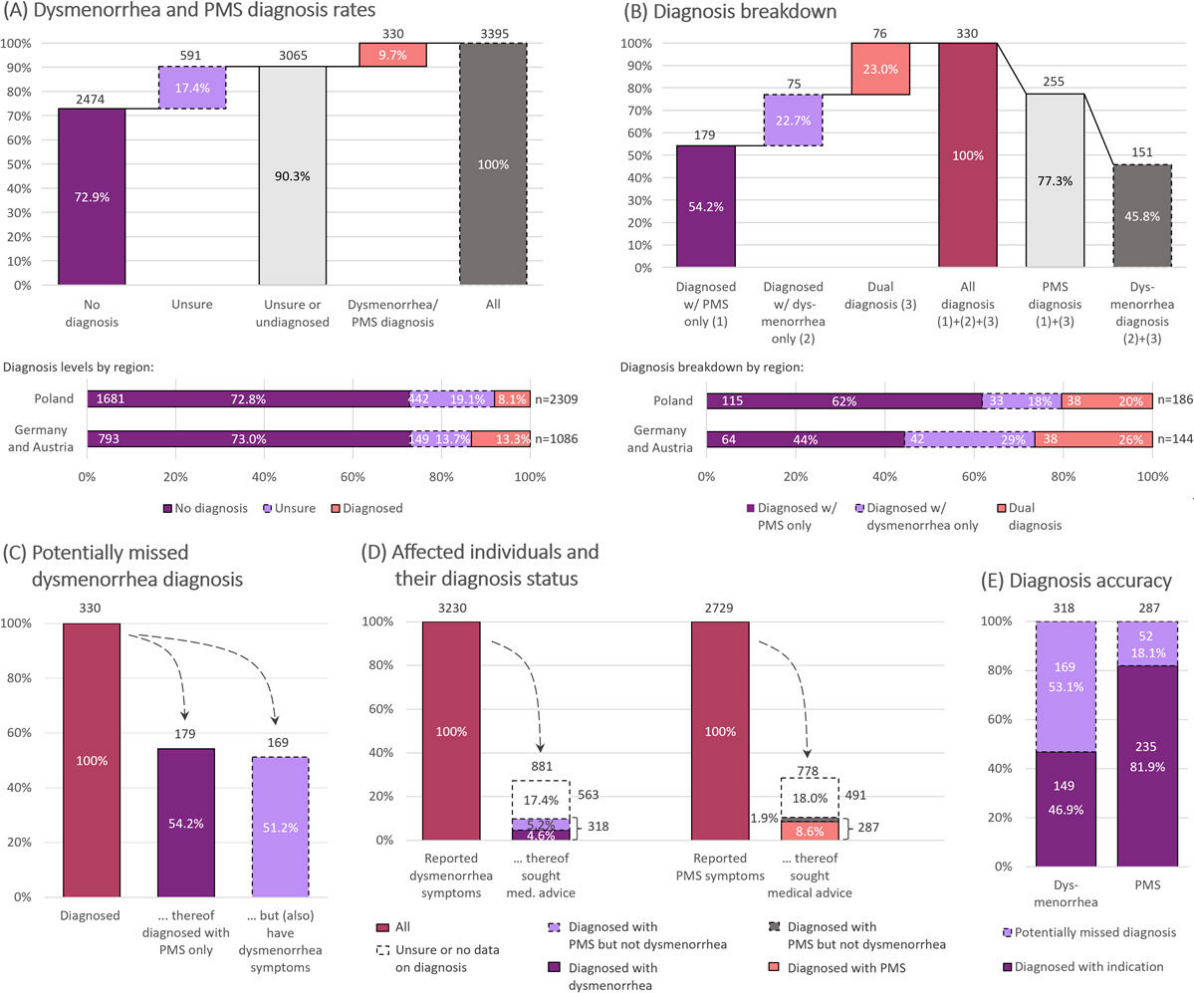
Dysmenorrhea and PMS diagnoses. (A) Dysmenorrhea and PMS diagnosis breakdown in the surveyed population (top) and its regional split (bottom). (B) Diagnosis breakdown for the 330 diagnosed women, showing the entire population (top) and regional splits (bottom). (C) Potentially missed dysmenorrhea diagnoses among the 330 diagnosed women. (D) Individuals with dysmenorrhea/PMS symptoms versus their diagnosis status; a mere 5%‐9% fraction is diagnosed with the respective indication. (E) Diagnosis accuracy by renormalizing the data from panel D to the number of individuals who reported a dysmenorrhea, PMS, or dual diagnosis. PMS: premenstrual syndrome.

Of all respondents to the menstrual pain campaign, 72.9% (2474/3395) were neither diagnosed with PMS nor dysmenorrhea by a medical doctor, regardless of being from Germany/Austria (793/1086, 73.0%) or Poland (1681/2309, 72.8%); see Figure 3A. In addition, 17.4% (591/3395; Germany/Austria: 149/1086, 13.7%; Poland: 442/2309, 19.1%) were unsure of their diagnosis status or chose not to disclose it in the anonymous questionnaire, reporting uncertainty about their diagnosis instead. The diagnosis rate remained unchanged when comparing women with lower pain levels (pain level <6: 226/248, 91.1% undiagnosed or unsure) to those with elevated pain levels (pain level ≥6: 2839/3153, 90.0% undiagnosed or unsure; see also Multimedia Appendix 3). This trend is consistent across both regions. Similarly, Cramer V revealed little to no association between elevated pain levels (V=0.040) and receiving a diagnosis of dysmenorrhea, PMS, or both. When considering only dysmenorrhea diagnoses, the correlation with pain levels was only marginally higher (V=0.055).

Of the 330 women who reported being diagnosed with dysmenorrhea, PMS, or both, 151 (45.8%) reported a dysmenorrhea diagnosis (n=75, 22.7% diagnosed solely with dysmenorrhea, plus n=76, 23.0% with a dual diagnosis of dysmenorrhea and PMS; see Figure 3B). Consistently, almost all (n=149) of the women diagnosed with dysmenorrhea (n=151) reported to regularly experience pain just before or during their period (1 day before and up to 3 days during menstrual bleeding), with none rating their strongest pain levels as merely 1 or 2 on a 0‐10 scale. Instead, 147 (97.4%) reported pain levels of 6 or higher, while only 4 individuals (2.6%) reported pain levels ranging from 3 to 5 (Can be found in Multimedia Appendix 3). Alongside those diagnosed with dysmenorrhea, 255 (77.3%) of the 330 diagnosed women received a PMS diagnosis. 179 (54.2%) of these individuals were diagnosed with PMS only, ie, without a concurrent dysmenorrhea diagnosis. However, of these 179 women, 169 (94.4%) still reported dysmenorrhea symptoms in the survey (Figure 3C).

### Diagnosis Situation of Women Experiencing Dysmenorrhea Symptoms

Of the 3230 women who reported symptoms suggestive of dysmenorrhea, 72.7% (2349) did not seek medical advice or their symptoms were not regarded as a pathological condition. A total of 318 individuals (9.8%) were diagnosed with either PMS or dysmenorrhea or both, while 17.4% (563/3230) reported being unsure of their diagnosis status (see Figure 3D). However, only 4.6% (149/3230) of the women experiencing symptoms related to dysmenorrhea also reported being diagnosed with the condition, with 7.7% (80/1041) in Germany/Austria and 3.2% (69/2189) in Poland. (In Poland, 2 individuals reported a diagnosis but not regular pain shortly before or during menstruation and are therefore excluded from this statistic.) Accordingly, the correlation between dysmenorrhea symptoms and having received a dysmenorrhea diagnosis is very weak (V=0.051). Even when considering only women experiencing pain levels of 6 or higher, the dysmenorrhea diagnosis level remained as low as 4.7% (146/3095). Notably, the remaining 5.2% (n=169) among the 9.8% (n=318) who received a diagnosis were diagnosed with PMS but not with dysmenorrhea, despite regularly experiencing symptoms related to dysmenorrhea. That is, among the 318 seemingly DYS-affected and diagnosed women, 169 (53.1%) remained undiagnosed with dysmenorrhea (Figure 3E).

### Diagnosis Rates of Women Experiencing PMS Symptoms

Similar to dysmenorrhea, most women (1951/2729, 71.5%) who reported symptoms suggestive of PMS either did not seek medical advice or were not diagnosed. Further, 10.5% (287/2729) reported a diagnosis of either PMS, dysmenorrhea, or both, while 18.0% (491/2729) were unsure of their diagnosis status. Among them, 8.6% (235/2729) reported being diagnosed with PMS, and 1.9% (52/2729) had dysmenorrhea but no PMS diagnosis (Figure 3D). The ratio of PMS diagnoses to potentially missed cases (diagnosed with dysmenorrhea but not PMS, yet reporting symptoms related to PMS in the questionnaire) is 80%:20% (235/287, or 81.9%, versus 52/287, or 18.1%; see Figure 3E). For completeness, we also report the correlation between PMS symptoms and a PMS diagnosis, which, though higher than for dysmenorrhea, is still low (V=0.090).

### Comorbidities

**Figure 4.**
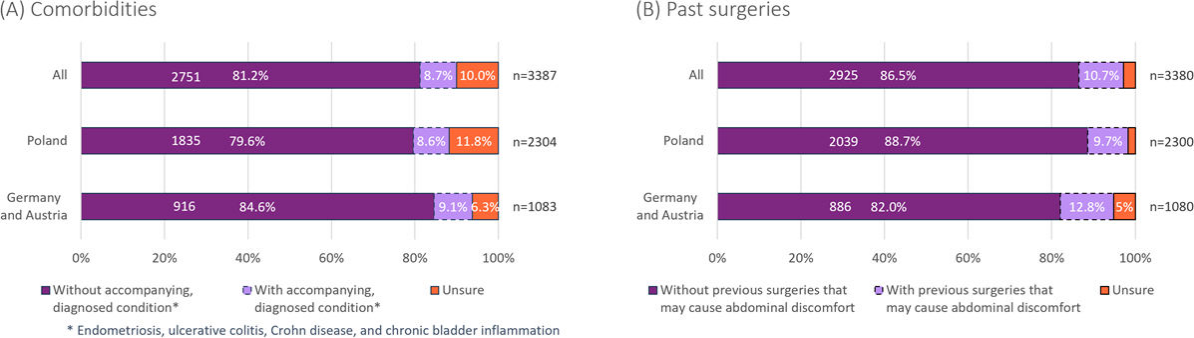
Comorbidities and surgery history. (A) Presence of comorbidities, specifically endometriosis, ulcerative colitis, Crohn disease, and chronic bladder inflammation, in the surveyed population. (B) History of surgeries that may cause abdominal discomfort or pain. The difference of 3387 versus 3380 for the total population is due to 7 individuals who stopped filling out the questionnaire between the 2 questions.

Primary dysmenorrhea was predominant in the surveyed population: 81.2% (2751/3387) had no comorbid conditions—such as endometriosis, ulcerative colitis, Crohn disease, or chronic bladder inflammation—and had not undergone surgeries that could cause abdominal discomfort (Figure 4A and B), while 8.7% (296/3387) stated that they were diagnosed with one of the aforementioned conditions. The questionnaire did not ask for additional indications. The fraction of individuals who underwent surgical procedures that may lead to painful conditions (eg, adhesions) or scarring in the lower abdomen was 10.7% (360/3380).

### Clinical Trial Eligibility and Willingness to Participate in a Clinical Trial

Roughly one-third (Germany/Austria: 245/639, 38.3%; Poland: 546/1529, 35.7%) of the surveyed women met all eligibility criteria for clinical trial participation (see Table 2). Of the participants, 64.7% (304/470) in Germany/Austria to 70.0% (670/957) in Poland are willing to use hormone-free contraception for 7 months in a clinical trial, already use hormone-free contraception, or are not sexually active at present. Almost none of the surveyed individuals had a history of cancer in the past 5 years. All other eligibility criteria are fulfilled by 80%-90% of women.

**Table 2.** Common criteria are crucial for clinical trial eligibility. The table follows a funnel-like structure: each row in the table incorporates the conditions of the preceding row and shows the number of individuals who meet the condition, relative to the total number of individuals evaluated at that step. The latter number is affected by participant dropouts occurring between questionnaire items.

	Germany and Austria, n/N (%)	Poland, n/N (%)
Survey participants who are aged 18–49, have a 24–38 day menstrual cycle, regularly experience pain of severity 5+ shortly before or during menstruation, and have had these symptoms since adolescence	639/639 (100)	1529/1529 (100)
Of those, who do not have gastrointestinal or urological conditions, or are unsure about it	573/639 (89.7)	1393/1529 (91.1)
Of those, who have no past surgeries causing ongoing pain, or are unsure about it	519/570 (91.1)	1275/1392 (91.6)
Of those, who are neither currently pregnant, trying to conceive, nor breastfeeding	471/519 (90.8)	957/1273 (75.2)
Of those, who are willing to use hormone-free contraception for 7 months during the clinical study, or are already using hormone-free contraception, or are not sexually active at present	304/470 (64.7)	670/957 (70.0)
Of those, who do not smoke or do not smoke daily	247/304 (81.3)	549/669 (82.1)
Of those, who have not had cancer in the last 5 years	245/246 (99.6)	546/549 (99.5)
Individuals who meet all of the above criteria	245/639 (38.3)	546/1529 (35.7)

**Figure 5.**
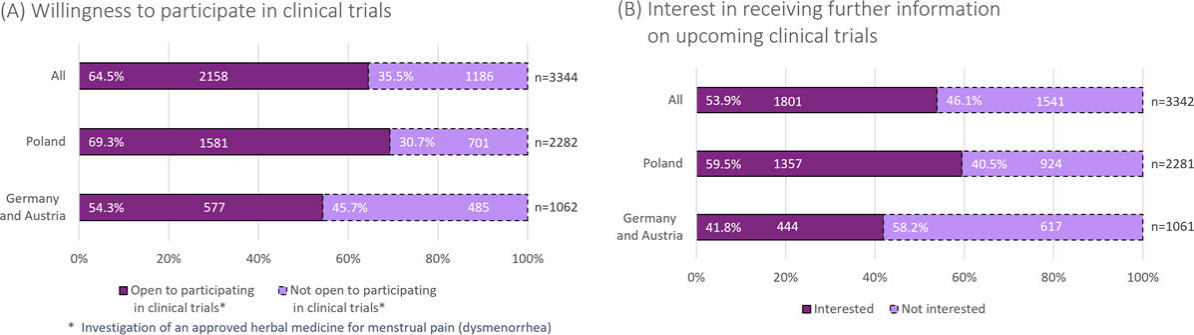
Openness to clinical trials. (A) Willingness to participate in a clinical trial on dysmenorrhea investigating an herbal medicine. (B) Interest in clinical trials is measured by the interest in receiving information about upcoming trials. The difference of 3344 versus 3342 for the total population is due to 2 individuals who stopped filling out the questionnaire between the 2 questions.

In Germany/Austria, 54.3% (577/1062) of the surveyed women responded positively regarding participation in a dysmenorrhea clinical trial that investigates an herbal medicine for menstrual pain and dysmenorrhea, and 41.8% (444/1061) were interested in further information. These numbers were higher in Poland: 69.3% (1581/2282) of respondents were open to participating in a clinical trial, and 59.5% (1357/2281) were interested in further information (Figure 5A and B). Consistent with somewhat higher pain levels in Poland, we found a weak-to-moderate association between higher pain levels and willingness to participate in clinical trials (V=0.150 for pain ≥6). As expected, a strong correlation was observed between willingness to participate in a clinical trial and interest in further information (V=0.477), subsequently triggering voluntary contact data sharing in the questionnaire.

### Cost Per Patient Reached in the Online Campaign

**Figure 6.**
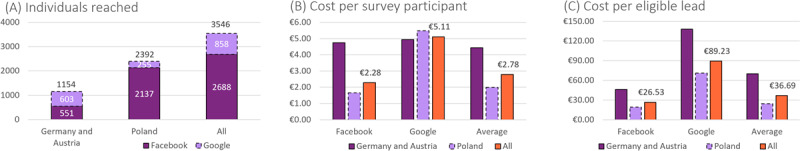
Cost efficiency. (A) Awareness creation, measured by the number of individuals reached. (B) Costs associated with awareness creation per individual reached. (C) Cost per eligible person, based on survey criteria and who shared their contact data, excluding donations. At the time of the study, €1 was equivalent to US $1.09.

In the 3 countries taken together, 3546 women (Germany/Austria: 1154; Poland: 2392) were reached in 14 days at marketing costs of €9869 (US $10,757; Germany/Austria: €5121 [US $5581.89]; Poland: €4748 [US $5175.32]), equal to more than 250 individuals per day at average costs of less than €3 (US $3.27) per person, using Facebook (Meta) and Google Ads to create awareness (Figure 6A and B). Accounting for donations totaling €6522 (US $7109; Germany/Austria: €2061 [US $2246.49]; Poland: €4461 [US $4862.49]) for fully completed questionnaires, the average total expense amounts to €4.62 (US $5.04) per individual reached via the online campaign. The awareness campaign was first launched and optimized in Germany/Austria, with the insights subsequently applied in Poland. Facebook proved to be more cost-effective than Google for raising awareness about the online questionnaire. With respect to the average cost per survey participant and monthly marketing budgets, the awareness campaign in Poland was more than twice as cost-effective and reached patients twice as quickly as compared to Germany/Austria: In Germany/Austria, we reached 82 individuals per day at average costs of €4.44 (US $4.84) per person, compared to Poland with 171 individuals per day at average costs of €1.99 (US $2.17) per person (donations not included). Differing efficiencies stemmed not only from regional disparities but also from (1) the optimization of Google and Facebook campaigns in Germany/Austria, which benefited the subsequent campaign in Poland from the very beginning, and (2) increased usage of Facebook over Google Ads in Poland.

Eligible leads—those with matching responses in the online questionnaire and who shared their contact details to receive updates on clinical trials against menstrual pain—were reached at average marketing campaign costs of €36.69 (US $39.99) per person (Figure 6C), excluding donations. When donations for completed questionnaires are included, total expenses increase by 66% (Germany/Austria: +40%, Poland: +94%), rising from €37 (US $40.33) to €61 (US $66.49) per eligible lead. Average marketing costs in Poland were about one-third of those in Germany/Austria. Like awareness creation costs, Facebook was notably more effective (€26.53, or US $28.92, per lead) than Google Ads (€89.23, or US $97.26, per lead).

## Discussion

### Apparent Prevalence, Pain Levels, and Intercorrelations Thereof

Of all surveyed women, 94.6% reported menstrual pain suggestive of dysmenorrhea. The high dysmenorrhea rate among the surveyed women demonstrates that our awareness creation campaigns reached the target population with high specificity. We emphasize that the campaign specificity should not be mistaken for being representative of the general population prevalence. The prevalence of dysmenorrhea ranges from 45% to 95% [3,5,27]. Advertisements primarily focused on menstrual pain (though they still addressed PMS), reducing the PMS specificity of the campaign. Coinciding with PMS prevalence [5,18,21], 80.1% of surveyed women experience PMS symptoms. Despite the high apparent prevalence of symptoms related to dysmenorrhea and symptoms related to PMS, the 2 indications are only weakly correlated in the surveyed population (V=0.100). Dysmenorrhea symptoms correlate with increased pain (V=0.299 for pain levels ≥6), whereas, as expected, symptoms related to PMS show little to no correlation with elevated pain (V=0.075 for pain levels ≥6). Quick et al [28] compared the QoL implications of primary dysmenorrhea with and without concurrent PMS. Irrespective of experiencing PMS, dysmenorrhea significantly reduced women’s QoL during the perimenstrual week. In addition, in this period, QoL was found to be slightly lower in the dysmenorrhea group with PMS compared to those with dysmenorrhea alone. Regarding pain levels in the surveyed population, we also observed slight regional differences: the average reported pain level in Poland, 8.4, exceeds that in Germany/Austria by 0.7 points. Irrespective of data uncertainty, lower pain levels in Germany/Austria compared to Poland might be due to higher usage of (hormonal) contraception in Germany/Austria, such as the oral contraceptive pill. In Germany/Austria, about 30.2% (349/1154) of the survey population used contraception, compared to 18.1% (433/2392) in Poland, according to this patient survey. The oral contraceptive pill may alleviate pain [4,29], making the treatment of dysmenorrhea a common (off-label) use for oral contraceptive pills [4,14,19]. The lower contraception rates in Poland compared to Germany/Austria are likely influenced by local cultural norms, historical context, the legal environment, and religious factors.

### Dysmenorrhea Underdiagnosis

Irrespective of pain levels, 90.3% (3065/3395) of the surveyed women did not seek medical advice, their symptoms were not regarded as a pathological condition, or they were unsure about their diagnosis (Figure 3A and Multimedia Appendix 3). This contrasts with the high fraction of women who reported symptoms related to dysmenorrhea (94.6%) or symptoms suggesting PMS (80.1%) (Figure 1A). The data suggest that women would rather accept discomfort than seek medical advice and that there may be a lack of sensitivity toward dysmenorrhea as a medical condition, at least in Germany, Austria, and Poland. Among the residual 9.7% (330/3395) of women who sought medical advice and received a diagnosis, about half were diagnosed with dysmenorrhea (Figure 3A and B). This diagnosis rate differs significantly from the 94.6% dysmenorrhea prevalence in the survey population (Figure 1A); that is, among those who sought medical advice, there is a significant gap between the realized diagnosis rate (149/318, 46.9%) and its statistical expectation (up to 94.6%). This situation leaves half of women (169 out of 318, or 53%) with a missed dysmenorrhea diagnosis even after they consulted a physician on their menstrual condition (Figure 3E) and ultimately results in a low dysmenorrhea diagnosis rate: less than 5% of women (4.6% in this survey) reporting dysmenorrhea symptoms receive a formal diagnosis (Figure 3D), despite high pain levels (Figure 2).

Diagnosis accuracy is notably different for PMS. The 77.3% (255/330) PMS diagnosis rate among women who sought medical advice (which in turn applies to 10.5% of all PMS-affected women; see Figure 3D) closely matches the statistical expectation, given the 80.1% PMS prevalence in the survey population (Figures3B  1B). While only half of dysmenorrhea-affected women who received a diagnosis on their menstrual condition ultimately received a dysmenorrhea diagnosis, about 80% of PMS-affected and diagnosed women (235/287, 81.9% in this survey) received a PMS diagnosis (Figure 3E). Overall, 8.6% of women with PMS symptoms were diagnosed with PMS—nearly twice the diagnosis rate of dysmenorrhea (4.6%).

Thus, when women seek medical advice, PMS appears to be diagnosed more accurately (and more often; see Figure 3B) than dysmenorrhea in the 3 countries included in the study. Given that dysmenorrhea and PMS symptoms may appear quite similar from the patients’ perspective, distinguishing between these 2 conditions becomes more challenging for patients. As a result, patients may perceive dysmenorrhea as part of PMS, attributing all symptoms, including pain, to PMS. Such a pattern is also reflected in online searches; there are about 90,500 Google searches for PMS per month in Germany in July 2024, compared to 12,100 for dysmenorrhea [30], suggesting that PMS receives nine times more attention (or is significantly more familiar to the public) than dysmenorrhea among those seeking information or advice. Mistaking dysmenorrhea for PMS may even lead some patients with a dysmenorrhea diagnosis to report a PMS diagnosis in the questionnaire instead. More importantly, this misperception might trigger the underdiagnosis of dysmenorrhea upon seeking medical advice: when consulting physicians, women may report being affected by PMS, while dysmenorrhea may not receive sufficient attention during the consultation. At the same time, the above figures suggest potentially reduced sensitivity among physicians towards dysmenorrhea and a possible lack of involvement from gynecologists.

### Online Recruitment Suited for Dysmenorrhea Clinical Trial Patient Enrollment

About one-third of individuals reached through Google Ads and Facebook were eligible for clinical trial participation, with more than half (Germany/Austria: 54.3%; Poland: 69.3%) open to participating, and similarly high interest in receiving further information. There’s an (intended) preselection bias, as women who are intrinsically interested in the topic are more likely to respond to the online ads and the subsequent survey, though only a weak-to-moderate association between higher pain levels and willingness to participate in clinical trials was found. For most individuals, this occurs alongside an undiagnosed condition (see above). Interested individuals were reached with high efficiency through the online campaign (3546 women reached in 14 days, at an average marketing cost of less than €3 [US $3.27] per person, excluding donations). These costs are notably lower than the typical range of €10 (US $11) to €25 (US $27) per person reached in online campaigns in Europe (SubjectWell empirical value, based on experience, for creating awareness using the above media), and are influenced by the indication, country, time of the year, and advertisement specifics, among others. High incidence, along with frequent, recurring pain, likely contributes to the high responsiveness to the online campaign, suggesting high efficiency in reaching out to people for clinical trial recruitment. Combined with high trial eligibility and high willingness to participate in clinical trials, qualified patient leads were reached at low costs (€37, or US $40, per eligible lead). For Bionorica’s subsequent clinical trial (European Union Clinical Trial number 2023-503688-41), online patient recruitment by SubjectWell (at that time: Trials24) was thus used in addition to traditional recruitment to ensure cost-efficient, on-schedule enrollments.

### Consistent Accompanying Data

Approximately 8.7% (296/3387) of surveyed women reported diagnoses of endometriosis, ulcerative colitis, Crohn disease, or chronic bladder inflammation. This aligns with the literature: around 10% of women with severe dysmenorrhea exhibit pelvic abnormalities, such as endometriosis or uterine anomalies [31]. The data are also consistent regarding smoking rates: 20.0% (231/1154) of participants from Germany/Austria are smokers, closely aligning with WHO data, which reports smoking rates of 19.6% for women in Germany and 22.9% for women in Austria [32,33]. In Poland, 18.6% (445/2392) of the survey participants are smokers, consistent with the WHO figure of 19.1% for women [34].

### Limitations

The results reported herein are subject to five limiting factors. First, raising awareness through an online campaign (Google Ads, Facebook) introduces a preselection bias, in this case by preferentially selecting individuals who react to and identify with menstrual pain. Consequently, prevalence estimates derived from this sample may not be representative of the general public but rather reflect the symptom distribution within the online-reached population. Similarly, the willingness to participate in clinical trials and interest in further information reported herein may reflect the campaign-targeted population rather than the general public. Second, individuals with higher pain levels may have been more inclined to start filling out the survey, potentially skewing the sample toward those with more severe symptoms and higher analgesic use. However, statistical analysis revealed no significant correlation between pain levels and survey completion. Third, all statements on medical conditions refer to phenomenological, patient-reported symptoms rather than clinical diagnoses; the terms “dysmenorrhea-affected” and “PMS-affected” are used for simplicity and do not imply a diagnosis; see also the Methods section. Fourth, regarding underdiagnosis in the surveyed population, two counteracting effects may be considered: (1) a sense of helplessness due to undiagnosed dysmenorrhea may have motivated participation, potentially resulting in an overestimation of missed diagnoses. (2) The sample may be biased toward individuals more willing to disclose symptoms, who may also be more likely to seek professional help, potentially leading to an underestimation of underdiagnosis. Fifth, we used the term “women” in some parts of the awareness campaign, which may have negatively affected gender diversity in the sample population. We acknowledge that not all individuals who menstruate identify as women.

### Conclusions

We examined two objectives: (1) assessing clinical and trial-relevant factors in women with dysmenorrhea or PMS beyond typical clinical reach, and (2) evaluating the effectiveness of online direct-to-patient outreach. We derived three key insights:

- 1A: Only a small fraction of women affected by dysmenorrhea or PMS are reached through clinical settings, for example, when relying on site-based clinical-trial recruitment only*.* Among the women we reached who reported symptoms related to dysmenorrhea, only 5% were diagnosed. Hence, 95% of the dysmenorrhea population we addressed is difficult to reach through conventional medical practices. For PMS, 9% were diagnosed. While these data rely on Germany, Austria, and Poland, we believe that most high-income and emerging countries exhibit similarly low diagnosis rates, and possibly even lower rates in low-income countries.
- 1B: Both low medical advice-seeking and missed dysmenorrhea diagnoses contribute to the overall low dysmenorrhea diagnosis rate. Up to 90% of affected women did not seek medical advice, were unsure about their diagnosis, or their symptoms were not regarded as a pathological condition. Of the 10% diagnosed in Germany, Austria, and Poland, half were diagnosed with dysmenorrhea, while the other half received solely a PMS diagnosis despite also experiencing dysmenorrhea symptoms. That is, the observed dysmenorrhea diagnosis rate (47%) falls short of the dysmenorrhea symptom rate in the surveyed population (95%). This situation is different for PMS: 77% of diagnosed women received a PMS diagnosis, in line with the expected rate of 80% in the surveyed population.
- 2: Online channels enabled efficient patient outreach paired with high patient engagement. In the 3 countries addressed, the online campaign reached over 250 individuals daily, totaling 3546 women, at a cost of less than €3 (US $3.27) per person (excluding donations; <€5 (US $5.45) when including donations). 95% of these women reported symptoms consistent with dysmenorrhea. Average costs per eligible lead were as low as €37 (US $40; excluding donations). The campaign and online questionnaire also achieved high patient engagement: Of the 3546 individuals, 94% completed all 18 health- or trial-related questions. Willingness to participate in a clinical trial was only weakly to moderately correlated with elevated pain levels.

For conditions where many individuals do not seek professional advice due to personal, societal, or condition-related reasons, direct-to-patient communication, leveraging online channels, can be key to engaging with them. The demonstrated effectiveness of online patient outreach is likely generalizable to a wide range of common diseases and regions, though testing on a case-by-case basis is recommended. For rare diseases, however, a more tailored “patient-by-patient” approach may be necessary. Broader patient reach can enhance patient diversity and improve access to clinical research (in this case, by avoiding the preselection bias of including only those 5% already diagnosed with dysmenorrhea), though demographic factors and recruitment through online versus offline channels [35] were not addressed in this study.

## Supplementary material

10.2196/68148 Multimedia Appendix 1 Facebook and Google campaign information.

10.2196/68148 Multimedia Appendix 2 Online questionnaire and its completion rates.

10.2196/68148 Multimedia Appendix 3 Supplementary data and analyses.

10.2196/68148 Multimedia Appendix 4 Survey data.

## Abbreviations

PII: personally identifiable information
PMS: premenstrual syndrome
QoL: quality of life
WHO: World Health Organization
