# Supplementary material for: Real-World Evidence Shows Gaps in Awareness, Medical Help-Seeking, and Diagnosis for Primary Dysmenorrhea but Not Premenstrual Syndrome: Cross-Sectional Observational Study
Source: J Med Internet Res. 2025 Sep 11;27:e68148. doi: 10.2196/68148 (PMC12425425; doi:10.2196/68148)
Supplement: Multimedia Appendix 1 [file jmir-v27-e68148-s001.docx]

Multimedia Appendix 1

Facebook and Google campaign information

supplementing the article

“Reaching out to patients: real-world evidence underscores lack of awareness and diagnosis for primary dysmenorrhea but not premenstrual syndrome (PMS)”

Matthias Roos, Verena Wimmelbacher, Lisa Klein, Marija Kesić, Ann-Katrin Rueß, Christina Necker,
Nicole Mähler, Petra Stute, Christoph Abels, and Tobias Kruse

# (A) Target group, visuals and texts used in the Facebook campaign

Facebook advertisements can be directed to a specific audience. The awareness campaign used these targeting options:

- Age: 18-49 years
- Location: up to 50 km radius around the planned study centers
- Gender: Women

**Figures A1.1** and **A1.2** show the advertisements displayed in the campaign in Germany/Austria and Poland, respectively.

| (a)  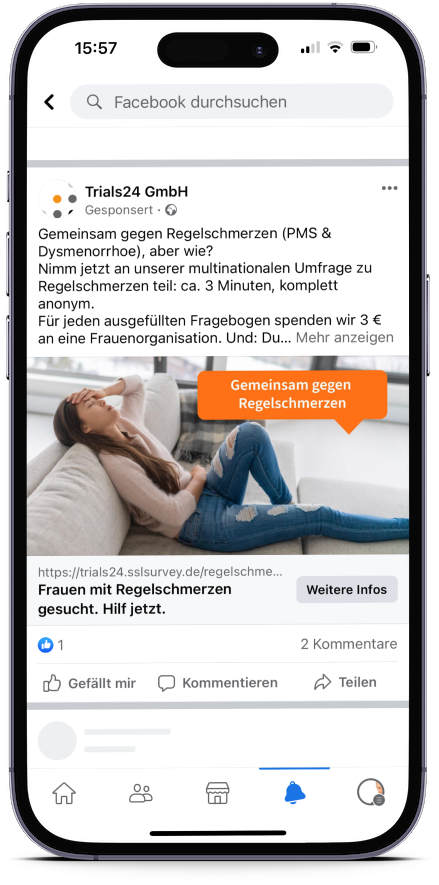 | (b)  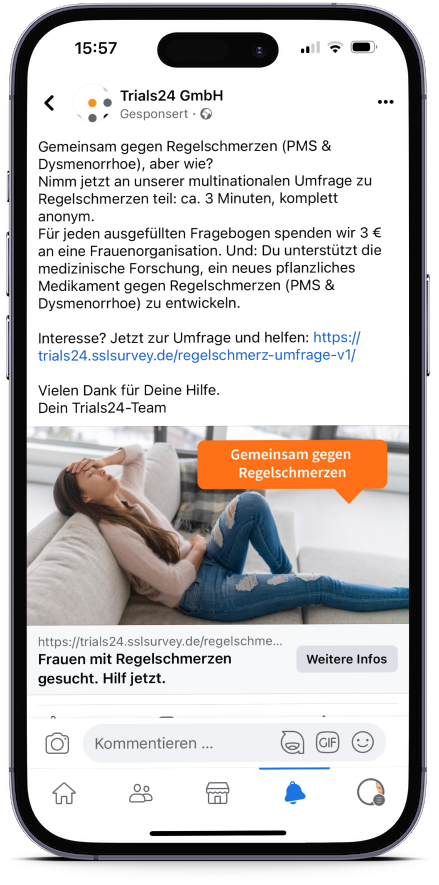 |
| --- | --- |

*Figure A1.1. Facebook advertisements in Germany and Austria. (a) Displayed as seen in users' news feeds. (b) Full text revealed after clicking "Show more" ("Mehr anzeigen").*

| (a)  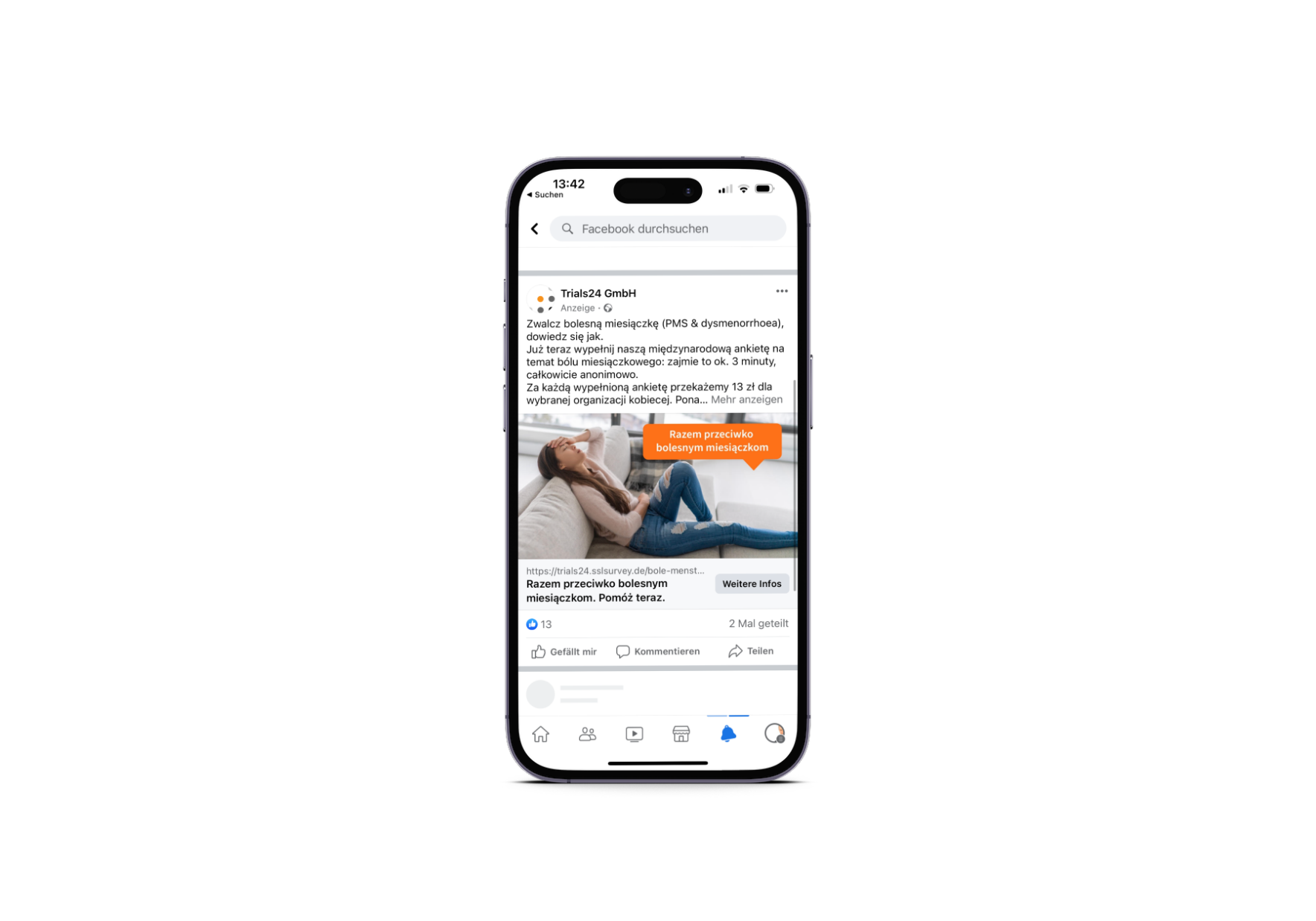 | (b)  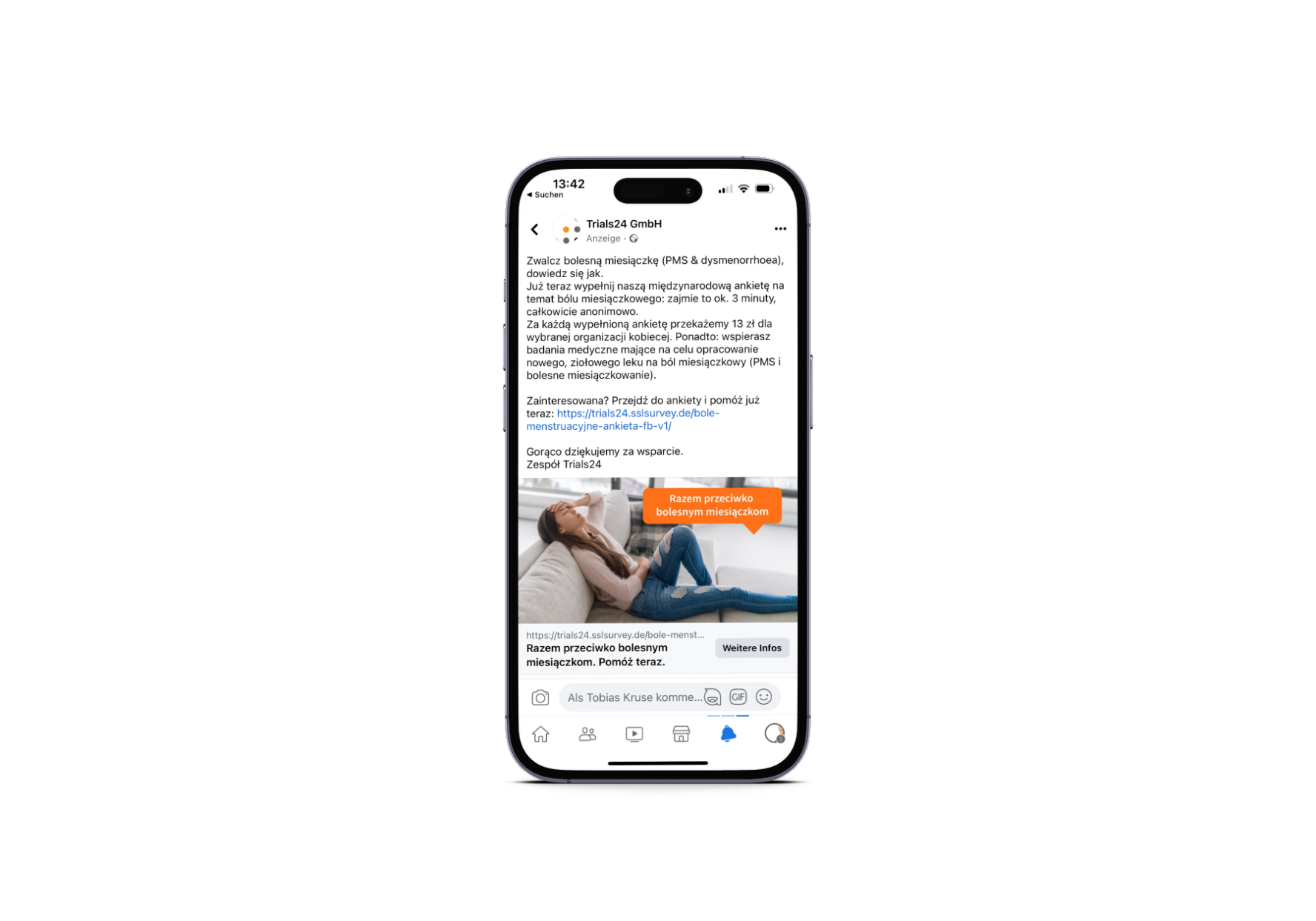 |
| --- | --- |

*Figure A1.2. Facebook advertisements in Poland. (a) Displayed as seen in users' News Feeds. (b) Full text revealed after clicking "Show more".*

# (B) Target group, visuals and texts used in the Google campaign

When a user enters the following search terms or queries into Google Search, advertisements for the awareness campaigns (**Figures A1.3** and **A1.4**) will appear above the search results:

- Dysmenorrhea
- PMS / premenstrual syndrome
- Period cramps / menstrual cramps
- Period pain / menstrual pain
- Painful period / painful menstruation

Advertisements were not displayed for search queries containing the following terms:

- Endometriosis
- PMDD / premenstrual dysphoric disorder

Google advertisements can target specific audiences, and the awareness campaign used the same targeting options as its Facebook counterpart:

- *Age:* 18-49 years
- *Location*: up to 50 km radius around study centers
- *Gender*: Women

| (a)  **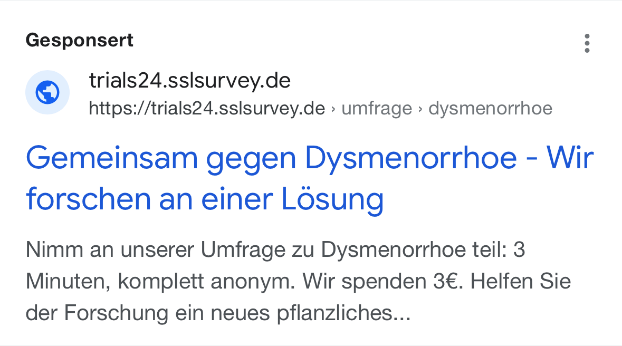** | (b)  **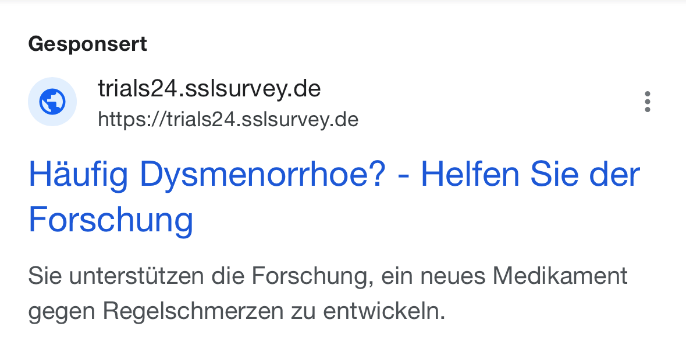** |
| --- | --- |

*Figure A1.3.* Google advertisements in Germany. *(a)* Together against dysmenorrhea – We are researching a solution. *(b)* Frequent dysmenorrhea – Support the research.


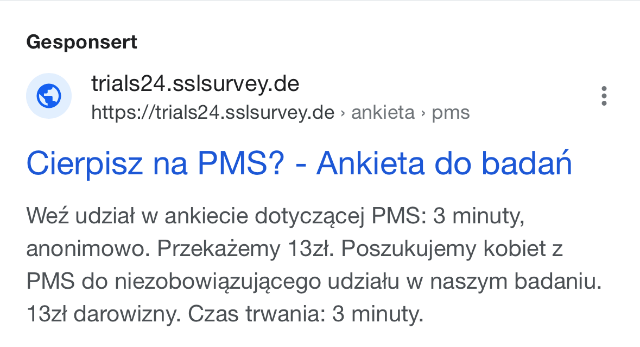


*Figure A1.4.* Google advertisements in Poland: Are you suffering from PMS? – Survey for research.
