# Supplementary material for: Real-World Evidence Shows Gaps in Awareness, Medical Help-Seeking, and Diagnosis for Primary Dysmenorrhea but Not Premenstrual Syndrome: Cross-Sectional Observational Study
Source: J Med Internet Res. 2025 Sep 11;27:e68148. doi: 10.2196/68148 (PMC12425425; doi:10.2196/68148)
Supplement: Multimedia Appendix 2 [file jmir-v27-e68148-s002.docx]

Multimedia Appendix 2

Online questionnaire and its completion rates

supplementing the article

“Reaching out to patients: real-world evidence underscores lack of awareness and diagnosis for primary dysmenorrhea but not premenstrual syndrome (PMS)”

Matthias Roos, Verena Wimmelbacher, Lisa Klein, Marija Kesić, Ann-Katrin Rueß, Christina Necker, Nicole Mähler, Petra Stute, Christoph Abels, and Tobias Kruse

# (A) Survey questionnaire

## The questionnaire was divided into four sections, featuring questions in the respondents' native languages (German and Polish). It included questions not discussed in the main article but relevant for assessing trial eligibility or contact patients for trial recruitment. For example, the age range of 18-49 years was relevant for assessing trial eligibility, though we did not restrict the analysis to this group, thereby also including younger individuals. 7% of participants were outside this age range. Below, we provide an English version for convenience. Native language versions are provided subsequently.

## Translation to English

### Section 1 – Introduction and questions about period pain (PMS & dysmenorrhea)

*Together against period pain (PMS & dysmenorrhea)*

Help medical research develop a new herbal medicine for period pain and participate in the multinational period pain survey (PMS & Dysmenorrhea) now. Please answer the following questions for us. It will take about 3 minutes and is anonymous. […]

*Thank you for your help – We are donating for your participation!*

With your response, you support medical research. In addition, for each completed survey, we will donate [€3 (Germany/Austria) / zł13 (Poland)] to a women’s organization that you can choose from a list at the end of the survey.

*Question 1:* Are you a woman between 18 and 49 years old?

- - Yes
  - No

*Question 2:* Does your menstrual cycle last between 24 and 38 days?

- - Yes
  - No
  - I am not sure

*Question 3:* Do you regularly experience pain 1 day before your period or within the first 3 days of your period?

- - Yes
  - No

*Question 4:* Do you regularly experience mood swings, pain or general discomfort 5 to 10 days before your period?

- - Yes
  - No

*Question 5:* How would you describe the strongest intensity of your period pain?

Please assess this according to your own perception.

- - Scale from 0 to 10 (0 = no pain or cramps, 10 = very severe pain or cramps)

*Question 6:* Did you experience period pain in your youth? (Up to 2 years after your first period)

- - Yes
  - No
  - I am not sure

*Question 7:* Has a doctor diagnosed you with PMS or dysmenorrhea?

- - Yes, only PMS
  - Yes, only dysmenorrhea
  - Yes, PMS and dysmenorrhea
  - No
  - I am not sure

*Question 8:* Do you take pain-relieving medication or other forms of medication against period pain?

- - Yes
  - No

*Question 9:* Have you been diagnosed with any of the following: endometriosis, ulcerative colitis, Crohn’s disease or chronic inflammation of the bladder?

- - Yes
  - No
  - I am not sure

*Question 10:* Have you undergone any operations in the past that are still causing pain, adhesions, or scarring in the lower abdomen?

- - Yes
  - No
  - I am not sure

*Question 11:* Are you trying to get pregnant, are you pregnant or are you breastfeeding?

- - Yes
  - No

*Question 12:* Are you currently using contraception?

- - Yes
  - No

***Remark****: The following question appeared only if the previous question is answered "Yes."*

*Question 13:* Which contraceptive do you currently use?

- - Hormonal (e.g., pill, vaginal ring, hormonal IUD, or similar).
  - Non-hormonal IUD (e.g. copper, gold)
  - Condom, diaphragm
  - Vasectomy of the partner or bilateral tubal occlusion
  - other methods

***Remark****: The following question was displayed only if question 12 was answered “No” or question 13 was answered “hormonal” or “non-hormonal IUD.”*

*Question 14:* Would you and your partner be willing to use a hormone-free contraceptive for up to 7 months as part of a clinical trial, if necessary?

For example: male or female condoms, sexual abstinence, diaphragm or a sponge with spermicide.

- - Yes
  - No
  - Not necessary. I don’t have a partner and am sexually abstinent

*Question 15:* Do you smoke every day?

- - Yes
  - No, I do not smoke
  - No, only occasionally (less than 1x a day)

*Question 16:* Have you had cancer in the last 5 years?

- - Yes
  - No

*Question 17:* Could you imagine, in principle, taking part in a clinical trial with an *approved herbal medicine* against period pain (dysmenorrhea)?

*Researchers are developing new herbal medicines for menstrual pain (dysmenorrhea), resulting in clinical trials requiring testing safety and efficacy. This often means finding many suitable patients with period pain. Only after the trials have been successfully completed will the medicines be available to all patients.*

*Important: Your answer to this question is completely non-binding for you.*

- - Yes
  - No

*Question 18:* Would you like to receive more information about new clinical trials for period pain, e.g., one with herbal medicine, as soon as participation is possible? […]

### Section 2 – More information for you

[…]

*Question 20*: The safety of your personal data is very important to us. Please give us your consent to send you the information.

- Yes, I agree to the processing of my data in accordance with the privacy policy [linked document] and confirm that I have read and accept it.

**Further answers**: Checkmark was actively ticked]

*Question 21:* Please leave your contact details:

[…]

### Section 3 – Feedback

Please help us collect vital information to reach other period pain patients better.

[…]

*Question 25:* How did you find out about the Period Pain Survey?

- - Google
  - Internet advertising
  - Facebook or Instagram
  - Family or acquaintance
  - Patient organization
  - Doctor
  - Other

### Section 4 – Thank you

Thank you for taking part in the Period Pain Survey!

[…]

*Question 26:* Please choose a women’s organization to donate to:

• […]

• […]

• […]

Thank you very much!

Your Trials24 Team

## Original language

The questionnaire items are presented below in their original languages (**Table A2.1**).

| ***Item*** | **Topic** | Questionnaire displayed in **Germany/Austria** | Questionnaire displayed in **Poland** |
| --- | --- | --- | --- |
| *Intro* | / | Gemeinsam gegen Regelschmerzen (PMS & Dysmenorrhoe)  Helfen Sie der medizinischen Forschung, ein neues pflanzliches Medikament gegen Regelschmerzen zu entwickeln, und nehmen Sie jetzt an der multinationalen Regelschmerz-Umfrage (PMS & Dysmenorrhoe) teil. Bitte beantworten Sie uns dazu die folgenden Fragen. Dies dauert ca. 3 Minuten und ist anonym. Alle Hintergrundinformationen zur Umfrage finden Sie hier [LINK zum PDF, öffnet in neuem Fenster].  Danke für Ihre Hilfe – wir spenden für Ihre Teilnahme!  Mit Ihrer Teilnahme unterstützen Sie die medizinische Forschung. Für jede vollständige Teilnahme an dieser Umfrage spenden wir 3€ an eine Frauenorganisation, die Sie am Ende der Umfrage aus einer Liste auswählen können. | Razem przeciwko bólom miesiączkowym (ZNP & dysmenorrhoea)  Pomóż badaniom klinicznym w opracowaniu nowego, ziołowego leku na bóle miesiączkowe biorąc udział w międzynarodowej ankiecie dotyczącym bólu miesiączkowego (ZNP & Dysmenorrhoea) już teraz. Prosimy o udzielenie odpowiedzi na poniższe pytania. Ankieta zajmie około 3 minuty i jest anonimowa.  Dziękujemy za pomoc - Przekazujemy darowiznę za udział!  Wypełniając ankietę wspierasz badania kliniczne. Dodatkowo, za każdą wypełnioną ankietę przekażemy 13 zł na rzecz organizacji kobiet, którą możesz wybrać z listy znajdującej się na końcu ankiety. |
| *Question 1* | Age, Sex | Sind Sie eine Frau zwischen 18 und 49 Jahren?   - Ja - Nein | Czy jesteś kobietą w wieku od 18 do 49 lat?   - Tak - Nie |
| *Question 2* | Menstrual cycle | Dauert bei Ihnen ein Menstruationszyklus zwischen 24 bis 38 Tage?   - Ja - Nein - Ich bin mir nicht sicher | Czy Pani cykl miesiączkowy trwa od 24 do 38 dni?   - Tak - Nie - Nie jestem pewna |

| *Question 3* | Pain during menstruation | Treten bei Ihnen regelmäßig Schmerzen kurz vor oder mit Ihrer Periode auf? (1 Tag vor und bis zu 3 Tage während der Blutung)   - Ja - Nein | Czy regularnie odczuwa Pani ból na 1 dzień przed miesiączką lub w ciągu pierwszych 3 dni miesiączki?   - Tak - Nie |
| --- | --- | --- | --- |
| *Question 4* | Discomfort/pain before menstruation | Treten bei Ihnen regelmäßig Schmerzen 5 bis 10 Tage vor Ihrer Periode auf?   - Ja - Nein | Czy regularnie doświadcza Pani wahań nastroju, bólu lub ogólnego dyskomfortu na 5 do 10 dni przed miesiączką?   - Tak - Nie |
| *Question 5* | Pain intensity | Wie würden Sie die stärkste Intensität Ihrer Regelschmerzen beschreiben?  Bitte beurteilen Sie dies nach Ihrem eigenen Empfinden.   - Skala von 0 bis 10  (0 = keine Schmerzen oder Krämpfe,  10 = sehr starke Schmerzen oder Krämpfe) | Jak określiłaby Pani najsilniejsze natężenie bólu miesiączkowego? Prosimy o ocenę wedle własnego odczucia.   - Skala od 0 do 10  (0 = brak bólu lub skurczów,  10 = bardzo silny ból lub skurcze) |
| *Question 6* | Menstrual Pain in youth | Traten Ihre Schmerzen bereits in Ihrer Jugend auf? (Bis zu 2 Jahre nach Ihrer ersten Periode)   - Ja - Nein - Ich bin mir nicht sicher | Czy w młodości doświadczyła Pani bólu miesiączkowego (do 2 lat od pierwszej miesiączki)?   - Tak - Nie - Nie jestem pewna |
| *Question 7* | PMS or dysmenorrhea diagnosis | Hat ein Arzt bei Ihnen PMS oder Dysmenorrhoe diagnostiziert?   - Ja, nur PMS - Ja, nur Dysmenorrhoe - Ja, PMS und Dysmenorrhoe - Nein - Ich bin mir nicht sicher | Czy lekarz zdiagnozował u Pani ZNP lub dysmenorrhoea?   - Tak, tylko ZNP - Tak, tylko dysmenorrhoea - Tak, ZNP i dysmenorrhoea - Nie - Nie jestem pewna |
| *Question 8* | Pain-relieving medication | Nehmen Sie schmerzlindernde Medikamente oder alternative Präparate gegen Ihre Regelschmerzen ein?   - Ja - Nein | Czy przyjmuje Pani leki przeciwbólowe lub inne na bóle miesiączkowe?   - Tak - Nie |
| *Question 9* | Accompanying diagnoses | Wurde bei Ihnen eine weitere gynäkologische oder gastrointestinale Erkrankung diagnostiziert?  Beispielweise: Endometriose, Myome, Polypen, Colitis, Morbus Crohn, Reizdarmsyndrom etc.   - Ja - Nein - Ich bin mir nicht sicher | Czy zdiagnozowano u Pani którekolwiek z następujących schorzeń: endometrioza, wrzodziejące zapalenie jelita grubego, choroba Crohna lub przewlekłe zapalenie pęcherza moczowego?   - Tak - Nie - Nie jestem pewna |
| *Question 10* | Surgeries | Hatten Sie in der Vergangenheit Operationen, die Ihnen heute Schmerzen, Verwachsungen oder Narbenbildung im Unterbauch verursachen könnten?   - Ja - Nein - Ich bin mir nicht sicher | Czy w przeszłości przeszła Pani jakieś operacje, które do dziś powodują ból, pozostawiły zrosty lub blizny w podbrzuszu?   - Tak - Nie - Nie jestem pewna |

| *Question 11* | Pregnancy | Versuchen Sie gerade schwanger zu werden, sind Sie schwanger, oder stillen Sie?   - Ja - Nein | Czy planuje Pani ciąże, lub jest w ciąży lub karmi piersią?   - Tak - Nie |
| --- | --- | --- | --- |
| *Question 12* | Contraception: current situation | Verhüten Sie gerade?   - Ja - Nein | Czy obecnie stosuje Pani antykoncepcję?   - Tak - Nie |
| *Question 13* | Contraception: type of | Welches Verhütungsmittel verwenden Sie derzeit?   - Hormonell (z.B. Pille, Vaginalring, Hormonspirale, oder ähnliche) - Nicht-hormonelle Spirale (z.B. Kupfer, Gold) - Präservativ oder Kondom, Diaphragma - Beidseitiger Eileiterverschluss oder Vasektomie des Partners - Andere Methoden | Jaki środek antykoncepcyjny stosuje Pani obecnie?   - Antykoncepcję hormonalną (np. pigułki, krążek dopochwowy, hormonalna wkładka wewnątrzmaciczna lub podobne) - Niehormonalna wkładka wewnątrzmaciczna (np. miedziana, złota) - Prezerwatywa, diafragma - Wazektomia partnera lub obustronna okluzja kanalików - Inne metody |
| *Question 14* | Contraception:  hormone-free | Wären Sie und Ihr Partner bereit, gegebenenfalls im Rahmen einer klinischen Studie ein hormonfreies Verhütungsmittel zu verwenden?  Beispielsweise: beidseitiger Eileiterverschluss, Vasektomie des Partners, sexuelle Abstinenz, männl./weibl. Kondom, Diaphragma, Schwamm mit Spermizid.   - Ja - Nein - Nein, ich habe keinen Partner und lebe sexuell abstinent | Czy Pani i Pani partner bylibyście gotowi stosować bezhormonalne środki antykoncepcyjne przez okres do 7 miesięcy w ramach badania klinicznego, jeśli byłoby to konieczne?  Na przykład: prezerwatywy męskie lub damskie, abstynencja seksualna, diafragma lub gąbka ze środkiem plemnikobójczym.   - Tak - Nie - Nie jest to konieczne.  Nie mam partnera i jestem abstynentką seksualną |
| *Question 15* | Smoking | Rauchen Sie täglich?   - Ja - Nein, ich bin Nichtraucherin - Nein, nur gelegentlich  (weniger als 1x am Tag) | Czy pali Pani codziennie papierosy?   - Tak - Nie, nie palę. - Nie, tylko sporadycznie  (mniej niż 1x dziennie) |
| *Question 16* | Past cancer | Hatten Sie in den letzten 5 Jahren Krebs?   - Ja - Nein | Czy w ciągu ostatnich 5 lat chorowała Pani na raka?   - Tak - Nie |

| *Question 17* | Willingness to participate in clinical trial | Könnten Sie sich grundsätzlich vorstellen,  an einer klinischen Studie teilzunehmen,  in denen ein zugelassenes pflanzliches Medikament gegen Dysmenorrhoe untersucht wird?  Forscher entwickeln neue pflanzliche Medikamente gegen Dysmenorrhoe. Hierbei müssen klinische Studien zur Prüfung der Sicherheit und Wirksamkeit durchgeführt werden. Dafür müssen oft viele geeignete Patientinnen mit Dysmenorrhoe gefunden werden. Erst nachdem die Studien erfolgreich abgeschlossen sind, stehen die Medikamente allen Patientinnen zur Verfügung.  Wichtig: Die Antwort auf die Frage ist für Sie völlig unverbindlich.   - Ja - Nein | Czy mógłaby Pani sobie wyobrazić udział w badaniu klinicznym z zatwierdzonym lekiem ziołowym przeciwko bólom miesiączkowym (dysmenorrhoea)?  Naukowcy opracowują nowe leki ziołowe na bóle menstruacyjne (dysmenorrhoea), dlatego też badania kliniczne mają za zadanie sprawdzenie bezpieczeństwa i skuteczności nowych leków. Często oznacza to konieczność znalezienia wielu odpowiednich pacjentek z bólami miesiączkowymi. Dopiero po pomyślnym zakończeniu badań leki te będą dostępne dla wszystkich pacjentek.  Ważne: Odpowiedź na to pytanie jest dla Pani całkowicie niewiążąca.   - Tak - Nie |
| --- | --- | --- | --- |
| *Question 18* | Interest in further info | Möchten Sie weitere Informationen zu neuen klinischen Studien gegen Regelschmerzen erhalten, z.B. mit einem pflanzlichen Medikament, sobald eine Teilnahme möglich ist?  Wichtig: Die Antwort auf die Frage ist für Sie völlig unverbindlich.   - Ja - Nein | Czy chciałaby Pani otrzymać więcej informacji o nowych badaniach klinicznych dotyczących bólu miesiączkowego, np. takich z zastosowaniem ziołolecznictwa, jak tylko udział w nich będzie możliwy?  Ważne: Odpowiedź na to pytanie jest dla Panicałkowicie niewiążąca.   - Tak - Nie |
| *Question 20* | Consent | Der Schutz Ihrer persönlichen Daten ist uns sehr wichtig. Bitte geben Sie uns Ihr Einverständnis, damit wir Ihnen die Informationen zusenden dürfen.   - Ja, ich bin mit der Verarbeitung meiner Daten gemäß der Datenschutzerklärung einverstanden und bestätige, dass ich diese gelesen habe und diese akzeptiere | Bezpieczeństwo Pani danych osobowych jest dla nas bardzo ważne. Prosimy o wyrażenie zgody na przesłanie informacji.   - Tak, wyrażam zgodę na przetwarzanie moich danych zgodnie z polityką prywatności i potwierdzam, że zapoznałam się z nią i ją akceptuję. |
| *Question 21* | Contact details | Hinterlassen Sie bitte Ihre Kontaktdaten: […] | Proszę pozostawić Pani dane kontaktowe:  […] |
| *Question 25* | Awareness creation (channel) | Wie haben Sie von der Regelschmerz-Umfrage erfahren?   - Google - Internet-Werbung - Facebook oder Instagram - Familie oder Bekannte - Patientenorganisation - Arzt - Sonstiges | Skąd dowiedziałaś się o badaniu dotyczącego bólu miesiączkowego?   - Google - Reklama internetowa - Facebook lub Instagram - Rodzina lub znajomi - Organizacja pacjentów - Lekarz - Inne |

## *Table A2.1. Survey questions in their original languages*

# (B) Eligibility criteria used to estimate general clinical trial eligibility

Eligibility criteria used to estimate clinical trial eligibility rates in Germany/Austria and Poland are displayed in **Table A2.2**.

| Question | 1 | 2 | 3*^,^ ** | 4*^,^ ** | 5 | 6 | 7 | 8* | 9 |
| --- | --- | --- | --- | --- | --- | --- | --- | --- | --- |
| Qualifying answer | Yes | Yes;  I am not sure | Yes; No | Yes; No | ≥ 5 | Yes | Yes (all types); I am not sure | Yes; No | No; I am not sure |

| Question | 10 | 11 | 12 | 13* | 14 | 15 | 16 | 17 | 18 |
| --- | --- | --- | --- | --- | --- | --- | --- | --- | --- |
| Qualifying answer | No; I am not sure | No | No | All answers | Yes | No; No, only occasionally (<1x a day) | No | Yes | Yes (for contact data sharing only) |

*Table A2.2. Eligible answers per survey question. (*) Questions that have no impact on clinical trial eligibility are marked with an asterisk. (**) Questions 3 and 4 are essential for identifying individuals affected by dysmenorrhea or PMS symptoms, where a “yes” response to either question qualifies the individual for consideration. Eligibility was assessed with respect to these individuals.*

# (C) Questionnaire completion rates and dropouts

Of the 3,546 individuals who opened the questionnaire, 3,342 (94.2%) completed all questions in section 1 (addressing their health situation, willingness to participate in a clinical trial, and interest in further information); cf. **Figure A2.1**. Most dropouts (98 out of 204, or 48% of all dropouts) occurred at question 1, meaning these individuals (2.8% of total) did not start filling out the questionnaire. Among individuals who began filling out the questionnaire by answering the first question, the completion rate reached 96.9% (3,342 out of 3,448). The dropout rate declined to 0.7% at the second question, then plateaued at 0.1% – 0.2% per question, except for the two questions on contraception. Notably, after completing the initial three questions, only 1 – 9 individuals dropped out per question, relative to the 3,340+ people who continued answering. Whether questions 13 and 14 (contraception) were displayed to the survey participants depended on their previous answers. By showing these questions on contraception to a subset of individuals only, questionnaire abandonment due to a sensitive question was reduced. Question 13 had 4 dropouts (out of 778 individuals who received the question), while Question 14 had 9 droupouts (out of 3036 individuals), totaling 13 dropouts for the two questions on contraception.


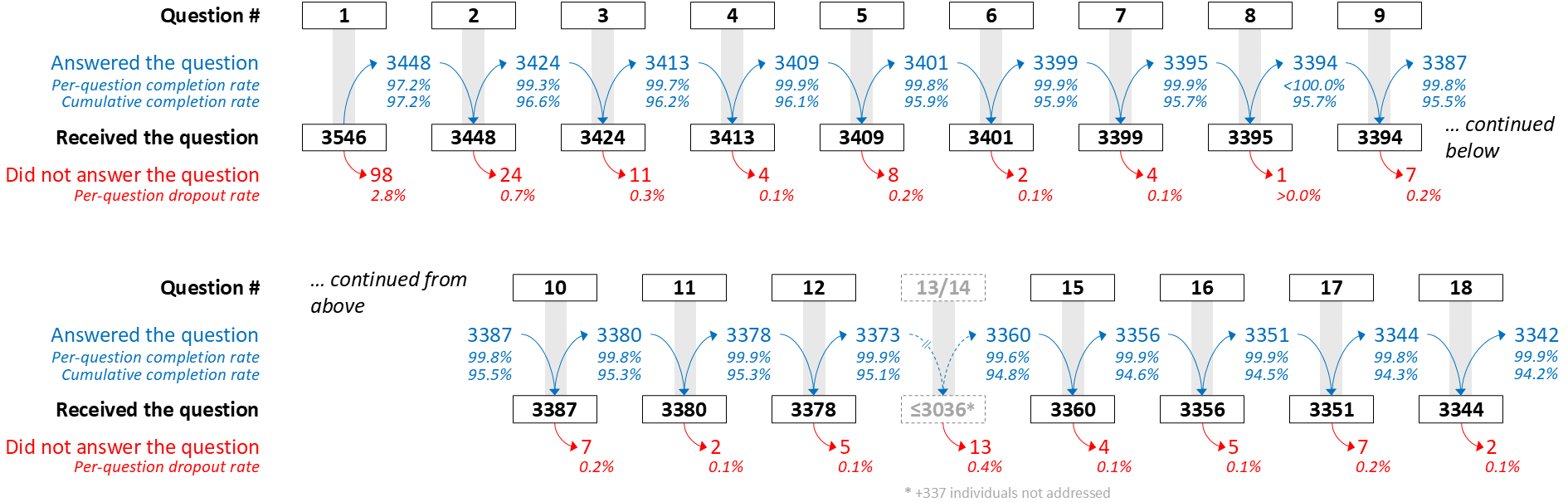


*Figure A2.1. Flowchart displaying, on a* *question-by-question basis, the fraction of individuals who answered each question versus those who stopped filling out the questionnaire (dropouts).
The cumulative completion rate is defined as the number of individuals who completed the questionnaire up to the given question relative to the total number who initially opened the questionnaire.*
