# Supplementary material for: Real-World Evidence Shows Gaps in Awareness, Medical Help-Seeking, and Diagnosis for Primary Dysmenorrhea but Not Premenstrual Syndrome: Cross-Sectional Observational Study
Source: J Med Internet Res. 2025 Sep 11;27:e68148. doi: 10.2196/68148 (PMC12425425; doi:10.2196/68148)
Supplement: Multimedia Appendix 3 [file jmir-v27-e68148-s003.docx]

Multimedia Appendix 3

Supplementary data and analyses

supplementing the article

“Reaching out to patients: real-world evidence underscores lack of awareness and diagnosis for primary dysmenorrhea but not premenstrual syndrome (PMS)”

Matthias Roos, Verena Wimmelbacher, Lisa Klein, Marija Kesić, Ann-Katrin Rueß, Christina Necker Nicole Mähler, Petra Stute, Christoph Abels, and Tobias Kruse

# (A) Cramer’s V: contingency tables and correlation strength

The data are presented in the order they appear in the main article; cf. **Tables A3.1 – A3.14**. The total number in each table corresponds to individuals who answered both questions.

|  | | **PMS symptoms** | | |  | **Cramer’s V correlation strength** |
| --- | --- | --- | --- | --- | --- | --- |
|  |  | **Yes** | **No** | **TOTAL** |  | 0.100  Weak |
| **Dysmenorrhea symptoms** | **Yes** | 2614 | 613 | 3227 | → |  |
|  | **No** | 115 | 67 | 182 |  |  |
|  | **TOTAL** | 2729 | 680 | 3409 |  |  |

*Table A3.1. dysmenorrhea vs. PMS symptoms.*

|  | | **Pain ≥ 6** | | |  | **Cramer’s V correlation strength** |
| --- | --- | --- | --- | --- | --- | --- |
|  |  | **Yes** | **No** | **TOTAL** |  | 0.299  Moderate |
| **Dysmenorrhea symptoms** | **Yes** | 3045 | 185 | 3230 | → |  |
|  | **No** | 108 | 75 | 183 |  |  |
|  | **TOTAL** | 3153 | 260 | 3413 |  |  |

*Table A3.2. dysmenorrhea symptoms vs. pain ≥ 6*

|  | | **Pain ≥ 6** | | |  | **Cramer’s V correlation strength** |
| --- | --- | --- | --- | --- | --- | --- |
|  |  | **Yes** | **No** | **TOTAL** |  | 0.075  None or very weak |
| **PMS symptoms** | **Yes** | 2551 | 178 | 2729 | → |  |
|  | **No** | 602 | 78 | 680 |  |  |
|  | **TOTAL** | 3153 | 256 | 3409 |  |  |

*Table A3.3. PMS symptoms vs. pain ≥ 6*

|  | | **Pain ≥ 8** | | |  | **Cramer’s V correlation strength** |
| --- | --- | --- | --- | --- | --- | --- |
|  |  | **Yes** | **No** | **TOTAL** |  | 0.176  Moderate |
| **Dysmenorrhea symptoms** | **Yes** | 2411 | 819 | 3230 | → |  |
|  | **No** | 73 | 110 | 183 |  |  |
|  | **TOTAL** | 2484 | 929 | 3413 |  |  |

*Table A3.4. dysmenorrhea symptoms vs. pain ≥ 8*

|  | | **Pain ≥ 8** | | |  | **Cramer’s V correlation strength** |
| --- | --- | --- | --- | --- | --- | --- |
|  |  | **Yes** | **No** | **TOTAL** |  | 0.083  None or very weak |
| **PMS symptoms** | **Yes** | 2039 | 690 | 2729 | → |  |
|  | **No** | 445 | 235 | 680 |  |  |
|  | **TOTAL** | 2484 | 925 | 3409 |  |  |

*Table A3.5. PMS symptoms vs. pain ≥ 8*

|  | | **Pain ≥ 6** | | |  | **Cramer’s V correlation strength** |
| --- | --- | --- | --- | --- | --- | --- |
|  |  | **Yes** | **No** | **TOTAL** |  | 0.352 Strong |
| **Pain medication usage** | **Yes** | 2831 | 108 | 2939 | → |  |
|  | **No** | 316 | 139 | 455 |  |  |
|  | **TOTAL** | 3147 | 247 | 3394 |  |  |

*Table A3.6. Pain medication usage vs. pain ≥ 6*

|  | | **Pain medication usage** | | |  | **Cramer’s V correlation strength** |
| --- | --- | --- | --- | --- | --- | --- |
|  |  | **Yes** | **No** | **TOTAL** |  | 0.213 Moderate |
| **Dysmenorrhea symptoms** | **Yes** | 2839 | 376 | 3215 | → |  |
|  | **No** | 100 | 79 | 179 |  |  |
|  | **TOTAL** | 2939 | 455 | 3394 |  |  |

*Table A3.7. dysmenorrhea symptoms vs. medication usage*

|  | | **Pain medication usage** | | |  | **Cramer’s V correlation strength** |
| --- | --- | --- | --- | --- | --- | --- |
|  |  | **Yes** | **No** | **TOTAL** |  | 0.057 None or very weak |
| **PMS symptoms** | **Yes** | 2381 | 338 | 2719 | → |  |
|  | **No** | 558 | 117 | 675 |  |  |
|  | **TOTAL** | 2939 | 455 | 3394 |  |  |

*Table A3.8. PMS symptoms vs. medication usage*

|  | | **Pain ≥ 6** | | |  | **Cramer’s V correlation strength** |
| --- | --- | --- | --- | --- | --- | --- |
|  |  | **Yes** | **No** | **TOTAL** |  | 0.040 None or very weak |
| **Any diagnosis (PMS/DYS)** | **Yes** | 309 | 21 | 330 | → |  |
|  | **Unsure** | 560 | 31 | 591 |  |  |
|  | **No** | 2279 | 195 | 2474 |  |  |
|  | **TOTAL** | 3148 | 247 | 3395 |  |  |

*Table A3.9. Pain ≥ 6 vs. dysmenorrhea/PMS diagnosis received*

|  | | **Pain ≥ 6** | | |  | **Cramer’s V correlation strength** |
| --- | --- | --- | --- | --- | --- | --- |
|  |  | **Yes** | **No** | **TOTAL** |  | 0.055 None or very weak |
| **Dysmenorrhea diagnosis** | **Yes** | 147 | 4 | 151 | → |  |
|  | **Unsure** | 560 | 31 | 591 |  |  |
|  | **No** | 2441 | 212 | 2653 |  |  |
|  | **TOTAL** | 3148 | 247 | 3395 |  |  |

*Table A3.10. Pain ≥ 6 vs. dysmenorrhea diagnosis received*

|  | | **Dysmenorrhea symptoms** | | |  | **Cramer’s V correlation strength** |
| --- | --- | --- | --- | --- | --- | --- |
|  |  | **Yes** | **No** | **TOTAL** |  | 0.051 None or very weak |
| **Dysmenorrhea diagnosis** | **Yes** | 149 | 2 | 151 | → |  |
|  | **Unsure** | 549 | 42 | 591 |  |  |
|  | **No** | 2518 | 135 | 2653 |  |  |
|  | **TOTAL** | 3216 | 179 | 3395 |  |  |

*Table A3.11. dysmenorrhea symptoms vs. dysmenorrhea diagnosis*

|  | | **PMS symptoms** | | |  | **Cramer’s V correlation strength** |
| --- | --- | --- | --- | --- | --- | --- |
|  |  | **Yes** | **No** | **TOTAL** |  | 0.090 None or very weak |
| **PMS diagnosis** | **Yes** | 235 | 20 | 255 | → |  |
|  | **Unsure** | 482 | 109 | 591 |  |  |
|  | **No** | 2003 | 546 | 2549 |  |  |
|  | **TOTAL** | 2720 | 675 | 3395 |  |  |

*Table* *A3.12. PMS symptoms vs. PMS diagnosis*

|  | | **Pain ≥ 6** | | |  | **Cramer’s V correlation strength** |
| --- | --- | --- | --- | --- | --- | --- |
|  |  | **Yes** | **No** | **TOTAL** |  | 0.150 Moderate [at border to being weak] |
| **Trial participation willingness** | **Yes** | 2063 | 95 | 2158 | → |  |
|  | **No** | 1037 | 149 | 1186 |  |  |
|  | **TOTAL** | 3100 | 244 | 3344 |  |  |

*Table A3.13. Pain ≥ 6 vs. willingness to participate in clinical trial*

|  | | **Trial participation willingness** | | |  | **Cramer’s V correlation strength** |
| --- | --- | --- | --- | --- | --- | --- |
|  |  | **Yes** | **No** | **TOTAL** |  | 0.477 Strong |
| **Interest in further information** | **Yes** | 1542 | 259 | 1801 | → |  |
|  | **No** | 614 | 927 | 1541 |  |  |
|  | **TOTAL** | 2156 | 1186 | 3342 |  |  |

*Table A3.14. Willingness to participate in clinical trial vs. interest in receiving further data (leading to contact data sharing)*

# (B) Diagnosis rates for pain levels above/below 6

Diagnosis rates remain essentially unchanged when comparing women with lower pain levels (pain level <6) to those with elevated pain levels (pain level ≥6); cf. **Table A3.15**. The numbers underlined are discussed in the main article.

|  | Germany/Austria | | | Poland | | All | | All, subgroups | |
| --- | --- | --- | --- | --- | --- | --- | --- | --- | --- |
| All pain levels | 1089 | | | 2312 | | 3401 | |  |  |
| Pain < 6 | 128 | 100% | | 120 | 100% | 248 | 100% |  |  |
| • No answer | 1 | |  | 0 |  | 1 |  |  |  |
| • Says to have no diagnosis | 104 | | 81.3% | 91 | 75.8% | 195 | 78.6% | 226 | 91.1% |
| • Unsure | 10 | | 7.8% | 21 | 17.5% | 31 | 12.5% |  |  |
| • Says to have diagnosis | 13 | | 10.2% | 8 | 6.7% | 21 | 8.5% | 21 | 8.5% |
| Pain ≥ 6 | 961 | 100% | | 2192 | 100% | 3153 | 100% |  |  |
| • No answer | 2 | |  | 3 |  | 5 |  |  |  |
| • Says to have no diagnosis | 689 | | 71.7% | 1,590 | 72.5% | 2279 | 72.3% | 2839 | 90.0% |
| • Unsure | 139 | | 14.5% | 421 | 19.2% | 560 | 17.8% |  |  |
| • Says to have diagnosis | 131 | | 13.6% | 178 | 8.1% | 309 | 9.8% | 309 | 9.8% |

*Table A3.15. Dysmenorrhea diagnosis rates. The numbers underlined are discussed in the main article.*

# (C) Subgroups of dysmenorrhea-diagnosed women

Of the 330 women aware of having dysmenorrhea, PMS, or both, 151 reported being diagnosed with dysmenorrhea. Among these 151 women, 149 regularly experience pain just before or during their period, and none rated their strongest pain levels below 3 on a scale from 0 to 10; see **Table A3.16**.

|  | Germany/Austria | | Poland | | All | |
| --- | --- | --- | --- | --- | --- | --- |
| Dysmenorrhea diagnosed | | | | | | |
| • Total | 80 | 100% | 71 | 100% | 151 | 100% |
| *- Subgroup:* Regularly experience pain just before or during the period* | | | | | | |
| • Yes | 80 | 100% | 69 | 97.2% | 149 | 98.7% |
| • No | 0 | 0% | 2 | 2.8% | 2 | 1.3% |
| *- Subgroup*: Pain levels | | | | | | |
| • Pain level <3 | 0 | 0% | 0 | 0% | 0 | 0% |
| • Pain level 3-5 | 3 | 3.8% | 1 | 1.4% | 4 | 2.6% |
| • Pain ≥6 | 77 | 96.3% | 70 | 98.6% | 147 | 97.4% |
| *- Subgroups*: Pain started in adolescence** | | | | | | |
| • Yes | 59 | 73.8% | 58 | 81.7% | 117 | 77.5% |
| • Unsure | 8 | 10.0% | 5 | 7.0% | 13 | 8.6% |
| • No | 13 | 16.3% | 8 | 11.3% | 21 | 13.9% |

* 1 day before and up to 3 days during the bleeding

** (up to 2 years after their first period)

*Table A3.16. Dysmenorrhea diagnosis subgroups. The numbers underlined are discussed in the main article.*
